# HEXB Drives Raised Paucimannosylation in Colorectal Cancer and Stratifies Patient Risk

**DOI:** 10.1016/j.mcpro.2025.100927

**Published:** 2025-02-11

**Authors:** Rebeca Kawahara, Liisa Kautto, Naaz Bansal, Priya Dipta, The Huong Chau, Benoit Liquet-Weiland, Seong Beom Ahn, Morten Thaysen-Andersen

**Affiliations:** 1 School of Natural Sciences, Macquarie University, Sydney, New South Wales, Australia; 2 Institute for Glyco-core Research (iGCORE), Nagoya University, Nagoya, Aichi, Japan; 3 School of Mathematical and Physical Sciences, Macquarie University, Sydney, New South Wales, Australia; 4 Université de Pau et Pays de L’Adour, Laboratoire de Mathématiques et de leurs Applications de PAU, CNRS, E2S-UPPA, Pau, France; 5 Macquarie Medical School, Faculty of Medicine, Health and Human Sciences, Macquarie University, Sydney, New South Wales, Australia

**Keywords:** colorectal cancer, glycoproteomics, glycosylation, HEXB, survival

## Abstract

Noninvasive prognostic markers are needed to improve the survival of colorectal cancer (CRC) patients. Toward this goal, we applied untargeted systems glycobiology approaches to snap-frozen and formalin-fixed paraffin-embedded tumor tissues and peripheral blood mononuclear cells from CRC patients spanning different disease stages and matching controls to faithfully uncover molecular changes associated with CRC. Quantitative glycomics and immunohistochemistry revealed that noncanonical paucimannosidic *N*-glycans are elevated in CRC tumors relative to normal adjacent tissues. Cell origin–focused glycoproteomics enabled using the well-curated Human Protein Atlas combined with immunohistochemistry of CRC tumor tissues recapitulated these findings and indicated that the paucimannosidic proteins were in part from tumor-infiltrating monocytes (*e*.*g*., MPO, AZU1) and of CRC cell origin (*e*.*g*., LGALS3BP, PSAP). Biosynthetically explaining these observations, *N*-acetyl-β-D-hexosaminidase (Hex) subunit β (*HEXB*) was found to be overexpressed in CRC tissues relative to normal adjacent colorectal tissues and colocalization and enzyme inhibition studies confirmed that HEXB facilitates paucimannosidic protein biosynthesis in CRC cells. Employing a sensitive, quick, and robust enzyme activity assay, we then showed that Hex activity was elevated in plasma and peripheral blood mononuclear cells from patients with advanced CRC relative to controls and those with early-stage disease. Surveying a large donor cohort, the plasma Hex activity was found to be raised in CRC patients relative to normal controls and correlated with the 5-year survival of CRC patients indicating that elevated plasma Hex activity is a potential disease risk marker for patient outcome. Our glycoproteomics-driven findings open avenues for better prognostication and disease risk stratification in CRC.

## Highlights

• Multiomics investigation of colorectal cancer specimens (tissues, PBMCs, plasma).
• Paucimannosidic proteins of immune and cancer origins dominate in CRC tumors.
• HEXB, a truncating glycoenzyme, drives paucimannosidic protein formation in CRC.
• Plasma hexosaminidase activity associates with 5-year survival of CRC patients.

## Graphical abstract

**Figure:**
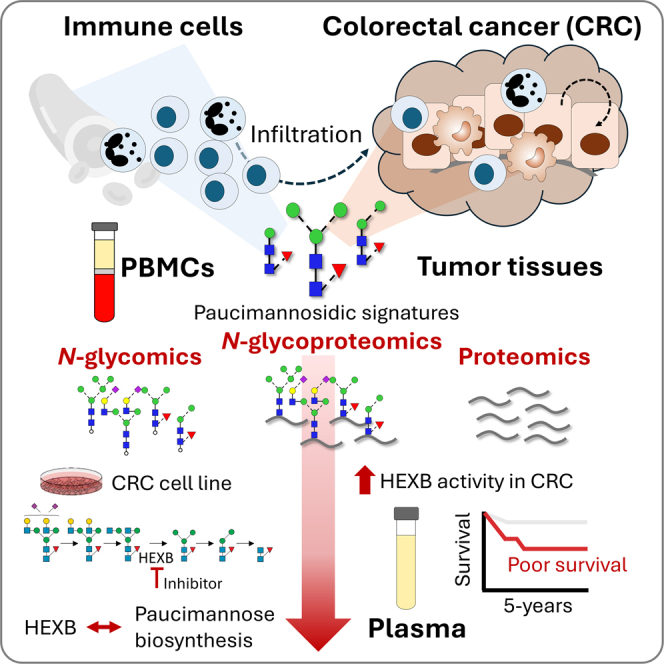

## In Brief

Aiming to find glyco-markers against colorectal cancer (CRC), we applied systems glycobiology to CRC patient specimens. Multiomics data revealed that noncanonical paucimannosidic proteins form prominent glyco-signatures in CRC tumors and that these originate from tumor-infiltrating monocytes and cancer cells. We then showed that *HEXB*, a truncating glyco-enzyme overexpressed in CRC tumors, facilitates paucimannosidic protein formation in CRC cells. Finally, plasma hexosaminidase activity was shown to associate with CRC patient survival. Our omics-guided findings open new avenues for effective CRC prognostication.

Colorectal cancer (CRC) is the second-leading cause of cancer-related deaths worldwide, accounting for approximately 1 million deaths annually (1). Currently, prognostication and treatment decisions are guided by the tumor-node-metastasis (TNM) staging system, determined subjectively through clinical and histopathological features of tissue biopsies at the time of diagnosis (2). Surgical removal is preferred for early and locally advanced CRC, while adjuvant chemotherapy is recommended if the CRC has already spread to adjacent lymph nodes.

CRC is a heterogeneous disease with variable outcomes even across cases with the same TNM stage (3). Most patients diagnosed with locoregional CRC will benefit from surgical resection of primary tumors. However, 30 to 40% of those patients will develop metastasis in the subsequent years, and the cure rates for this large patient group are dishearteningly low (4). Although the detection of genetic mutations in KRAS, APC, and TP53 in CRC tissues is currently used to predict response to therapy and patient risk (survival chances), the invasive nature and costs of such genetic analyses limit their clinical utility (5). Thus, there is a need for novel noninvasive prognostic markers that can identify CRC patients with high risk of poor outcome, thereby providing opportunities for early and more aggressive intervention and consequently improved patient survival while avoiding unnecessary overtreatment of low-risk patient groups (6).

Aberrant asparagine (*N*)-linked glycosylation has repeatedly been linked to malignant transformation and tumor progression in CRC (7, 8, 9) and across other cancers (10, 11, 12), thus offering a considerable and still largely untapped potential for biomarker discovery and therapeutic applications. However, glycoproteins are analytically challenging to study within complex cellular and tissue environments even in specialized biochemical laboratories due to the inherent glycoproteome heterogeneity and their unpredictable structural characteristics arising from nontemplated biosynthetic processes (13). While altered *N*-glycosylation has previously been reported in tumor tissues (TUMs) and sera of CRC patients (14, 15, 16, 17) and was found to hold a potential to inform on drug resistance (18), prognosis (19), staging (20), and recurrence (16) in CRC, the lack of biomarker specificity and the need for sophisticated glyco-analytical workflows have hitherto precluded the translation of these findings into the clinic.

Aiming to discover robust and clinically compatible (easy-to-assay) glyco-markers to aid the management and outcomes of CRC patients, in this work, we first applied untargeted systems glycobiology approaches to both snap-frozen and formalin-fixed paraffin-embedded (FFPE) TUMs as well as peripheral blood mononuclear cells (PBMCs) from cohorts of CRC patients spanning different disease stages (I–IV) and matching controls to comprehensively characterize molecular aberrations occurring in the glycoproteome during disease. We found that noncanonical paucimannosidic glycans some of which we suggest are carried by proteins of tumor-infiltrating monocyte and cancer cell origins form prominent glyco-signatures in CRC TUMs. We then showed that gene encoding the *N*-acetyl-β-D-hexosaminidase (Hex) subunit β (*HEXB*) is overexpressed in CRC TUMs and used a potent enzyme inhibitor to show that HEXB facilitates paucimannosidic protein formation in CRC cells. Finally, we document that the Hex activity in neat plasma is elevated in advanced CRC and stratifies patient risk in terms of their 5-year survival outcome. Collectively, our omics-guided findings open new avenues for effective prognostication in patients suffering from CRC.

## Experimental Procedures

### CRC and Donor Biospecimens

The study was approved by the Human Research Ethics Committee (Medical Sciences) at Macquarie University, Sydney, Australia (Protocol 5201800073) and abides by the Declaration of Helsinki principles. All biospecimens including snap-frozen and FFPE tissues, PBMCs, and plasma samples were sourced from the Victorian Cancer Biobank (VCB). Paired sets of snap-frozen tissues, PBMCs and plasma were collected from 28 patients who were clinically diagnosed with CRC and their disease progression was staged (I–IV) by trained pathologists at the VCB according to the TNM staging system (n = 7/stage). In addition to the CRC TUMs, seven paired normal adjacent tissues (NATs) and one unpaired NAT (all snap-frozen from CRC patients in stage I) were used as tissue controls (n = 8). Furthermore, FFPE sections of paired or unpaired TUM and NAT (thickness: 5 μm/slide, size: 2 cm × 2 cm) were obtained from CRC patients in stage I, II, and III. EDTA plasma from 78 healthy donors and 302 CRC patients for whom 5-year survival outcome posttreatment/surgery and other key metadata were known were obtained from the VCB with consent from all relevant parties. All biospecimens, clinical details, technical approaches, overview of raw data, and data availability are summarized in Supplemental Tables S1–S3.

### LIM2405 Cell Cultures

The CRC patient–derived cell line LIM2405 (CVCL_4437, adenocarcinoma of the caecum of a male CRC patient) was cultured in RPMI-Hepes with 2 mM glutamine (Sigma) supplemented with 10% (v/v) fetal bovine serum (Invitrogen), 1% (v/v) penicillin-streptomycin (Thermo), 1 μg/ml hydrocortisone (Sigma), 0.025 U/ml insulin (Sigma), and 0.01 μg/ml thioglycerol (Sigma) at 37 °C in humidified atmosphere containing 5% (v/v) CO_2_. Mycoplasma-free LIM2405 cells were seeded (10^5^ cells/ml) in RPMI media into 6-well plates (Corning) and incubated for 2 days at 37 °C in 5% (v/v) CO_2_. After cells reached confluency, RPMI media was added containing different concentrations of M31850 in dimethyl sulfoxide (DMSO) (0 μM, 0.24 μM, 7.81 μM, 15.62 μM, and 31.25 μM) or different concentrations of DMSO alone (0 μM, 0.24 μM, 7.81 μM, 15.62 μM, and 31.25 μM). All cells were incubated with M31850 or vehicle (DMSO) for 24 h, 37 °C.

### Cell Viability Assay

LIM2405 cells (5 × 10^4^ cells/ml) were seeded into 96-well microplates (Greiner 655160, tissue culture grade, flat bottom) in 100 μl culture media without Hex inhibitor (vehicle only) or with Hex inhibitors (M31850 in DMSO) spanning an extended concentration range (0.24, 7.81, 15.62, 31.25, 62.50, and 125 μM). Cells were incubated for 24 h at 37 °C in 5% (v/v) CO_2_ and 10 μl 0.5 mg/ml 3-(4,5-dimethylthiazol-2-yl)-2,5-diphenyltetrazolium bromide reagent (final concentration) was added to each well. Cells were incubated for another 3 h at 37 °C, 5% (v/v) CO_2_. After removing the media, 100 μl solubilization reagent containing 40% (v/v) dimethylformamide, 2% (v/v) glacial acetic acid, and 16% (w/v) SDS) (pH 4.7) was added to each well and the plate incubated for 15 min at 20 °C with gentle agitation. Absorbance of the formazan product was measured with Fluostar Galaxy (BMG) at 450 nm. The viability of Hex inhibitor-treated cells was determined in technical triplicates based on absorbance readings relative to untreated cells (vehicle) after subtracting the absorbance measured in blank samples.

### Protein Extraction

Snap-frozen tissues were thawed, lysed, and homogenized in a lysis buffer containing 8 M urea in 50 mM triethylammonium bicarbonate (TEAB) (pH 8.5) and a protease inhibitor cocktail (Roche) using zirconium beads (3 mm diameter, Sigma) in a TissueLyser (30 Hz, 2 min, Qiagen). Protein concentrations were determined by the bicinchoninic acid (BCA) method (Thermo).

To ensure sufficient sample material for downstream analyses, protein extracts from FFPE tissue slides from three different CRC patients or three slides from the same patients were pooled for each investigated CRC stage (stage I, II, and III). The FFPE tissues were manually scraped off the slides using a clean scalpel and the detached tissues were collected and pooled in microcentrifuge tubes for deparaffinization and protein extraction as described (21). Briefly, tissues were incubated with neat xylene for 1 min, vortexed for 1 min, and centrifuged at 12,000*g* for 15 min at room temperature (RT) to remove paraffin. The deparaffinized pellet was then rehydrated with 1 ml neat ethanol for 30 s followed by centrifugation at 12,000*g* for 15 min at RT. The tissue pellet was dried using a vacuum concentrator and resuspended in 300 μl lysis buffer containing 0.1 M dithiothreitol (DTT), 1 x protease inhibitor cocktail (complete ULTRA Tablets Mini, EDTA-free, Roche; 1:10), pH 8.5 and 4% (w/v) SDS (all final concentrations). Tissue lysis was performed for 1 h at 99 °C with slow agitation (400 rpm). Following lysis, samples were cooled to RT, and the tissue lysates were centrifuged for 30 min at 15,000*g* at 4 °C. Supernatants containing the protein extract were transferred to new microtubes. Protein concentrations were determined by BCA (Thermo) ahead of protein precipitation using four volumes of acetone (16 h, −30 °C). Proteins were pelleted (14,000 rpm, 10 min, 4 °C), and resuspended in 8 M urea in 50 mM TEAB.

PBMCs were thawed in serum-free RPMI medium, pelleted (500 g, 8 min, 20 °C), washed with 1 ml PBS, and centrifuged as above. The cell pellet was resuspended in 200 μl RIPA buffer containing PBS, 1% (v/v) Triton, 0.5% (w/v) sodium deoxycholate, 0.1% (w/v) SDS, and a protease inhibitor cocktail (Roche). Cells were lysed on ice for 30 min followed by sonication using a Branson 450 Digital Sonifier (two cycles of 5–10 s, 30% output, on ice). Cell debris was pelleted (10,000 rpm, 10 min, 4 °C) and supernatants containing the protein extract were transferred to new microtubes. Protein concentrations were determined by BCA (Thermo) ahead of protein precipitation using four volumes of acetone (16 h, −30 °C). Proteins were pelleted (14,000 rpm, 10 min, 4 °C), and resuspended in 8 M urea in 50 mM TEAB.

LIM2405 cells cultured with or without Hex inhibition were detached from culture plates with trypsin-EDTA (Sigma), washed with PBS, and lysed with RIPA buffer. Protein concentrations were determined by BCA. Proteins (50 μg/sample) were precipitated using cold acetone for 16 h at −30 °C. Samples were clarified by centrifugation (14,000 rpm, 10 min, 4 °C). The pellet was resuspended in 8 M urea and 50 mM TEAB.

### Protein Preparation for Glycomics and Glyco/proteomics

In preparation for the multiomics experiments, protein extracts from the snap-frozen and FFPE tissues, PBMCs, and LIM2405 cell line samples (50 μg/sample) were reduced using 10 mM DTT (30 min, 30 °C) and alkylated using 40 mM iodoacetamide (final concentrations, 30 min, in the dark, 20 °C). Alkylation reactions were quenched using excess DTT. Samples were split setting aside 15 μg protein extract for glycomics (see below) and 35 μg protein extract for glyco/proteomics. For the latter, samples were digested using sequencing grade porcine trypsin (1:50, w/w; 12 h, 37 °C, Promega). Proteolysis was stopped by acidification using 1% (v/v) TFA (final concentration). Peptides were desalted using primed Oligo R3 reversed-phase solid phase extraction (SPE) microcolumns as described (22) and dried.

### Glycan Preparation for Glycomics

In preparation for glycomics, proteins (15 μg/sample) were immobilized on a primed 0.45 μm polyvinylidene fluoride membrane (Merck Millipore) and handled as described (23). Briefly, *N*-glycans were released using 10 U recombinant *Elizabethkingia miricola N*-glycosidase F (10 U/μl, 16 h, 37 °C, Promega). Detached glycans were reduced in 1 M sodium borohydride in 50 mM potassium hydroxide (3 h, 50 °C). Reactions were stopped using glacial acetic acid, and glycans were desalted using strong cation exchange/C18 and porous graphitized carbon (PGC) SPE microcolumns.

### TMT Labeling of Peptides

Peptides from the snap-frozen and FFPE tissues, PBMCs, and LIM2405 cell line samples (35 μg/sample) were labeled with tandem mass tags (TMTs). Separate peptide reference pools were generated for the tissue and PBMC samples by pooling peptides from all those samples to enable quantitative comparisons across multiple TMT-10plex experiments. The individual peptide samples were then randomly combined across four TMT-10plex sets for both the tissue and PBMC samples, see Supplemental Table S2 for design. Each set included at least one sample from each CRC stage and a control sample. The 126 Da reporter ion was consistently used as the reference channel. For both the tissue and PBMC experiments, a total of 36 peptide samples were labeled with TMT including seven samples from each CRC stage and eight controls.

For each sample, peptides from 25 μg protein extract were dissolved in 100 μl 100 mM TEAB and labeled with TMT10plex tags (0.23 mg in 41 μl neat anhydrous acetonitrile (ACN), 1 h, 20 °C, Thermo). Labeling reactions were quenched using 8 μl 5% (v/v) hydroxylamine (15 min, 20 °C). Labeled peptides were mixed 1:1 (w/w) and desalted using hydrophilic–lipophilic balance SPE cartridges (Waters). A small aliquot containing 10 μg peptide mixture was set aside for direct liquid chromatography - tandem mass spectrometry (LC-MS/MS) analysis (unenriched fraction) and dried. The remaining samples were dried for glycopeptide enrichment ahead of downstream LC-MS/MS.

### Glycopeptide Enrichment

TMT-labeled peptide mixtures (∼240 μg combined) were reconstituted in 50 μl 80% ACN in 1% (both v/v) TFA and loaded onto primed custom-made hydrophilic interaction liquid chromatography (HILIC) SPE microcolumns packed with zwitterionic ZIC-HILIC resin (10 μm particle size, 200 Å pore size, kindly provided by Merck Millipore) onto supporting C8 disks (Empore) in p10 pipette tips as described (22, 24). Briefly, the flow-through/wash fractions containing nonglycosylated peptides were collected for separate downstream analysis, and the retained glycopeptides were eluted over three rounds with 0.1% (v/v) TFA, 25 mM ammonium bicarbonate, and then 50% (v/v) ACN. Eluted fractions were pooled. The unenriched and HILIC flow-through (peptides) and enriched (glycopeptides) fractions were separately desalted on primed Oligo R3 reversed-phase SPE microcolumns, aliquoted, and dried.

### High pH Prefractionation of Glyco/peptides

The peptide and glycopeptide fractions were resuspended in 50 μl 25 mM ammonium bicarbonate and separately loaded onto primed Oligo R2 reversed-phase SPE microcolumns packed on supporting C18 discs (Empore) in p10 pipette tips for high pH prefractionation as described (22, 24). Briefly, following several thorough washing steps, the glyco/peptides were sequentially eluted with 25 mM ammonium bicarbonate in 10% ACN (fraction 1), 25 mM ammonium bicarbonate in 20% ACN (fraction 2), and 25 mM ammonium bicarbonate in 60% ACN (fraction 3), dried, and resuspended in 0.1% (v/v) formic acid (FA) for separate LC–MS/MS analysis.

### LC-MS/MS

For glycomics, the released *N*-glycans were separated on an UltiMate 3000 HPLC system (Dionex) interfaced with a LTQ Velos Pro linear ion trap mass spectrometer (Thermo). The glycans were loaded on a PGC HPLC capillary column (Hypercarb KAPPA, 5 μm particle size, 200 Å pore size, 180 μm inner diameter × 100 mm length, Thermo), operated at 50 °C with a constant flow rate (4 μl/min) and supplemented with a post-column make-up flow supplying pure ACN at 4 μl/min delivered by the HPLC system (25). The mobile phases were ammonium bicarbonate (10 mM), pH 8.0 (solvent A), and 10 mM ammonium bicarbonate in 70% (v/v) ACN (solvent B) employing a 86 min-gradient: 8 min at 2.6% B, 2.6 to 13.5% B over 2 min, 13.5 to 37.3% B over 55 min, 37 to 64% B over 10 min, 64 to 98% B over 1 min, 5 min at 98% B, 98-2.6% B over 1 min, and 4 min at 2.6% B. The electrospray ionization source was operated in negative ion polarity mode with a source potential of 3.6 kV. Full MS1 scans (*m/z* 570–2000) were acquired using one microscan, *m/z* 0.25 full width half maximum (FWHM) resolution, 5 × 10^4^ automatic gain control (AGC), and 50 ms maximum accumulation time. MS/MS data were acquired using *m/z* 0.35 FWHM resolution, 2 × 10^4^ AGC, 300 ms maximum accumulation time, and 2 *m/z* precursor ion isolation window. The five most abundant precursors were selected from each MS1 full scan for collision-induced dissociation–based MS/MS using a normalized collision energy of 33% with an activation Q of 0.250 and 10 ms activation time.

For glyco/proteomics, TMT-labeled glyco/peptide mixtures were loaded on a trap column (2 cm × 100 μm inner diameter) custom-packed with ReproSil-Pur C18 AQ 5 μm resin ID (Dr Maisch) and separated on an analytical column (Reprosil-Pur C18-Aq; 25 cm × 75 μm, 3 μm ID, Dr Maisch) at 300 nl/min provided by an UltiMate 3000 RSLCnano HPLC system. The mobile phases were 99.9% ACN in 0.1% (both v/v) FA (solvent B) and 0.1% (v/v) FA (solvent A). The gradient was 2 to 30% B over 100 min, 30 to 50% B over 18 min, 50 to 95% B over 1 min, and 9 min at 95% B. The nanoLC was connected to a Q-Exactive HF-X Hybrid Quadrupole-Orbitrap mass spectrometer (Thermo) operating in positive ion polarity mode. The Orbitrap acquired full MS1 scans (*m/z* 350–1,800, AGC: 3 × 10^6^, 50 ms maximum accumulation, 60,000 FWHM resolution at *m/z* 200). Employing data-dependent acquisition, the 20 most abundant precursor ions from each MS1 full scan were isolated and fragmented utilizing higher-energy collision-induced dissociation (HCD, normalized collision energy 35%). Only multicharged precursors (Z ≥ 2) were selected for fragmentation. Fragment spectra were acquired in the Orbitrap (45,000 resolution, AGC: 1 × 10^5^, 90 ms maximum accumulation, *m/z* 1.0 precursor isolation window, 30 s dynamic exclusion after a single isolation/fragmentation of a given precursor).

### LC-MS/MS Data Processing and Analysis

Xcalibur (v2.2, Thermo) was used to inspect, browse, and annotate the raw LC–MS/MS data.

For glycomics, the glycans were manually identified and quantified as described (24, 26, 27). Briefly, glycan precursor ions were extracted using RawMeat (v2.1, Vast Scientific), common contaminants/redundant precursors were removed, and generic monosaccharide compositions (Hex, HexNAc, dHex, and NeuAc) were proposed using GlycoMod (http://www.expasy.ch/tools/glycomod). The glycan fine structures were manually elucidated using monoisotopic mass, PGC LC elution time, and MS/MS fragmentation pattern. Byos (v5.5.39, Protein Metrics) was used to annotate glycan fragment spectra and GlycoWorkbench 2 was used to generate cartoons of identified glycan structures. Glycans were quantified using extracted ion chromatograms in Skyline (v9.1, https://skyline.ms/) as described (27).

For glycoproteomics, HCD-MS/MS data of glycopeptides were searched with Byonic (v2.6.46, Protein Metrics) using 10/20 ppm as the precursor/product ion mass tolerance, respectively. Cys carbamidomethylation (+57.021 Da) and N-term/Lys TMT (+229.163 Da) were considered fixed modifications. Fully tryptic peptides were searched with up to two missed cleavages per peptide. The following variable modifications were selected allowing two common and one rare modification per peptide: Met oxidation (+15.994 Da, common) and *N*-glycosylation of sequon-localized Asn (rare) employing a glycomics-informed *N*-glycan search space (22). HexNAc_1_, HexNAc_1_Fuc_1_, and HexNAc_2_ were manually added to the glycan search space that comprised 74 and 57 *N*-glycan compositions shown with glycomics to exist in the investigated CRC tissue and PBMC, respectively. For LIM2405 cell line glycopeptide data was searched using a predefined *N*-glycan database of 309 mammalian *N*-glycans without sodium adducts available within Byonic to which the paucimannose M3F (Man_3_GlcNAc_2_Fuc_1_) was manually added. The HCD-MS/MS data were searched against all human proteins (20,300 sequences, UniProtKB, released December 11, 2019). All searches were filtered to <1% false discovery rate (FDR) at the glycoprotein level and 0% FDR at the glycopeptide level using a protein decoy database. Only confident glycopeptide identifications (PEP 2D < 0.001) were considered, and the search output was scrutinized; obviously wrong annotations were manually removed (24, 28). Glycopeptides were quantified using the “report ion quantifier” available as a node in Proteome Discoverer (v2.2, Thermo) as described (24). Glycopeptides were manually grouped by summing the reporter ion intensities from the glycopeptide-to-spectrum matches (glycoPSMs) belonging to the same UniProtKB identifier, same glycosylation site within the protein, and same glycan composition. Using TMT reporter ion intensities, unique glycopeptides (from grouped glycoPSMs) from each channel were first normalized against the reference channel (to allow data from multiple TMT batches to be combined) and further normalized within each channel (to correct for unintended channel variation introduced during labeling and mixing of samples).

For proteomics, HCD-MS/MS data (from both unenriched and HILIC flow-through fractions) were processed using MaxQuant (v1.6.12.0, https://www.maxquant.org) and searched against all human proteins (20,300 sequences, UniProtKB released December 11, 2019) using the Andromeda search engine (29) with a mass tolerance of 4.5 ppm for precursor ions and 20 ppm for product ions. The enzyme specificity was set to trypsin with a maximum of two missed cleavages permitted. Carbamidomethylation of Cys (+57.021 Da) and N-term/Lys TMT (+229.163 Da) were considered fixed modifications. Met oxidation (+15.994 Da) and protein N-terminal acetylation (+42.010 Da) were considered variable modifications. Both the protein and peptide level identifications were filtered to 1% FDR. Processing and statistical analyses (see below) of the MaxQuant output were performed in Perseus v2.0.7.0 (https://maxquant.net/perseus/). Proteins identified using the reverse database, proteins only identified through modified peptides, and proteins identified from the MaxQuant contaminant database were removed. By enabling the report fragment ions “10plex TMT” in the quantification settings, proteins were quantified by their reporter ion intensities using at least one razor/unique peptide per protein. The TMT-based protein abundances were normalized as described above. For comparison of *N*-acetyl-β-D-hexosaminidase subunit α (HEXA) and HEXB protein levels within samples, the reporter ion intensities were normalized only within each channel.

### Immunohistochemistry

Immunohistochemistry (IHC) was performed on paired FFPE tissue slides from CRC TUMs and NATs from a CRC patient in stage II using a published method (30) with some modifications. Briefly, FFPE slides were heated to 50 °C for 40 min followed by three cycles of deparaffination in neat xylene (30 min/cycle, 20 °C) and rehydration in 70% ethanol (3 min/cycle, 20 °C). Deparaffinated tissues were washed in Tris-buffered saline (TBS) and antigens were heat-retrieved in a Tris-EDTA buffer containing 10 mM Tris base, 1 mM EDTA, 0.05% (w/v) Tween20, pH 9.0 (95 °C, 30 min). Slides were cooled, washed with TBS, and blocked with 5% (w/v) bovine serum albumin (BSA, 99.9% (w/w) purity, 1 h, 20 °C, Sigma) to limit unspecific antibody binding. Importantly, BSA was pretreated with 100 mM sodium periodate (4 °C, 2 h) and dialyzed overnight in TBS to eliminate glycoepitopes from any contaminating glycoproteins. The BSA-blocked slides were washed with TBS and then stained with antibody. A fluorescence-conjugated rabbit monoclonal anti-human IgG-based antibody targeting the epithelial cellular adhesion molecule (EpCAM) was used to stain epithelial cells (anti-EpCAM-Alexa Fluor 647 (AF647), 1:100 dilution, 1 h, 20 °C, Abcam). To stain monocyte-derived anti-inflammatory macrophages known to express CD163 (31), a rabbit monoclonal anti-human CD163 IgG-based antibody (1:100 dilution, 16 h, 4 °C, Abcam) was applied, which was then visualized using a fluorophore-labeled goat monoclonal anti-rabbit IgG-Alexa fluor 405 IgG (1:400 dilution, 1 h, 20 °C, Abcam). As described (32, 33), paucimannosidic glyco-epitopes were detected using a mouse IgM antibody “Mannitou” kindly provided by Prof Simone Diestel, Univ Bonn (1:2 dilution of supernatant collected from cultured hybridomas, 2 h, 20 °C). Mannitou was visualized using a monoclonal anti-mouse IgM produced in goat (1:400 dilution, 1 h, 20 °C, Thermo) conjugated with Alexa fluor 488. The tissue slides were washed four times between each of the antibody staining steps with warm TBS containing 0.5% (w/v) Tween20 (5 min/washing step) followed by a final TBS rinse for 5 min. To preserve fluorescence, coverslips were mounted to the tissue sections using antifade mounting media (Thermo). The tissue stains were visualized, and colocalization was qualitatively assessed using an OlympusFV-3000 confocal microscope with 40x and 60x magnification using oil immersion objectives. Image data were handled using cellSens imaging software (Olympus). Quantification of Mannitou intensity (proportion of the total area) was performed using Image J (https://imagej.net/ij/).

### Gene and Protein Expression Profile and Cell Annotation

Gene expression (mRNA) data of *HEXA* and *HEXB* from colon (COAD) and rectum (READ) TUMs and matched NAT were retrieved from the Cancer Genome Atlas and the Genotype-Tissue Expression portal and interrogated in the Gene Expression Profiling Interactive Analysis platform. Significance cutoff was set to log2 fold change <0.5 and adjusted *p* < 0.001. Data were displayed by boxplots generated in the Gene Expression Profiling Interactive Analysis platform (34).

The relative tissue expression of HEXA and HEXB protein from paired TUM and NAT was determined by reinterrogation of quantitative proteomics data sourced from the Clinical Proteomic Tumor Analysis Consortium. The quantitative (TMT-based) proteomics data that can be accessed through https://linkedomics.org/cptac-colon/ were sourced from Vasaikar *et al*. (35). In contrast, the relative levels of HEXA and HEXB protein in nondepleted plasma from CRC patients (pretreatment group) and healthy controls were determined by reinterrogation of quantitative proteomics data collected using a data-independent acquisition strategy by Li *et al*., (36).

The Human Protein Atlas (HPA) was leveraged to propose cellular origin(s) of proteins identified in the snap-frozen TUMs and PBMCs (37, 38). Proteins that were found to be altered in the tissue proteomics data were mapped against entries in the tissue section of the HPA and those mapping to bone marrow–annotated proteins (534 proteins) were suggested to arise from immune cell sources. Similarly, altered proteins in the PBMC proteomics data were mapped against the entries in the blood section of the HPA suggesting proteins arising from natural killer (NK) cells (114 proteins), B cells (141 proteins), T cells (262 proteins) and monocytes (201 proteins).

### Hex Activity Assay

Hex enzyme activity was established in LIM2405 (2 μg protein extract), PBMCs (1.5 μg protein extract), and in neat plasma (2 μl/sample). Samples were incubated in 30 μl prewarmed 3 mM 4-methylumbelliferyl-2-acetamido-2-deoxy-β-D-glucopyranoside (MUG) or 4-methylumbelliferyl-2-acetamido-2-deoxy-β-D-glucopyranoside-6-sulfate (MUGS) substrate (Merck Millipore) in phosphate–citrate buffer (pH 5.0, 37 °C, 30 min, in the dark) as described (39). Reactions were stopped by the addition of 200 μl 0.25 M glycine carbonate buffer (pH 10.0). Hex activity was measured through fluorometric quantitation of 4-methylumbelliferone detected by a plate reader (FLUOstar Optima, BMG Technologies) at excitation 360 nm and emission 450 nm using a 4-methylumbelliferone standard curve (Merck Millipore). The Hex activity was determined in technical triplicates and normalized within each plate assay based on control samples. Three independent experiments (n = 3) were performed for LIM2405 cells treated with 31.25 μM M31850 in DMSO or with 31.25 μM DMSO only. Hex activity was measured across 36 PBMC samples and 380 plasma samples (see Supplemental Table S3 for overview).

### Evaluation of the Robustness of the Hex Activity Assay

The robustness of the Hex activity assay was explored using blood from healthy volunteers collected by venipuncture into either empty (for serum) or EDTA- or heparin-containing (for plasma) collection tubes (Vacutainer, Becton Dickinson Co). Samples were stored for 20 min, 20 °C and plasma/serum isolated by centrifugation (1500*g*, 15 min, 20 °C). Samples were aliquoted and stored for either 4 h, 24 h, or 96 h at 20 °C, 4 °C, −20 °C, or −80 °C until analyzed for Hex activity as per above. The impact of freeze/thaw cycles was also determined.

### Experimental Design and Statistical Rationale

The discovery phase of the study was performed using snap-frozen tissues and PBMCs from a cohort of 36 individuals, which provides sufficient statistical power to detect glycosylation changes and associations across multi-omics datasets. Multiomics measurements of FFPE tissue sections of 28 individuals and a patient-derived CRC cell lime (LIM2405) were used to support observations. A large cohort of 380 plasma samples was used to evaluate the association of plasma Hex activity with clinical parameters and patient survival. Pathway enrichment analysis was performed using Database for Annotation, Visualization, and Integrated Discovery Bioinformatics (40) (National Institute of Health) using adjusted *p* < 0.05 as significance threshold. Bubble graphs and correlation plots were performed using SRplot (41). Associations were explored using Pearson correlation coefficients and tested for significance using *t* distribution tests with *p* < 0.05 employed as confidence threshold. For the -omics datasets and functional assays, significance was assessed by unpaired two-tailed student's *t*-tests or ANOVA adjusted for multiple comparisons using Tukey test with 0.05 used as the significance threshold (95% confidence interval) if not otherwise explicitly stated. Five-year survival analysis was performed using Kaplan–Meier survival plots and log-rank tests (*p* < 0.05). GraphPad Prism (v9.4.1, Dotmatics, https://www.graphpad.com/) or Perseus v2.0.7.0 (https://maxquant.net/perseus/) were used for statistical analysis. Cox proportional hazards model performed in the R software (https://www.r-project.org/) was used to determine associations between survival, age, and plasma Hex activity. If not mentioned otherwise, data are plotted as mean (average) and error bars indicate SD. Replicates (biological/technical) and statistical significance have been stated in the figure legends.

## Results

### Paucimannose Levels are Raised in TUMs and PBMCs from CRC Patients

**Figure 1:**
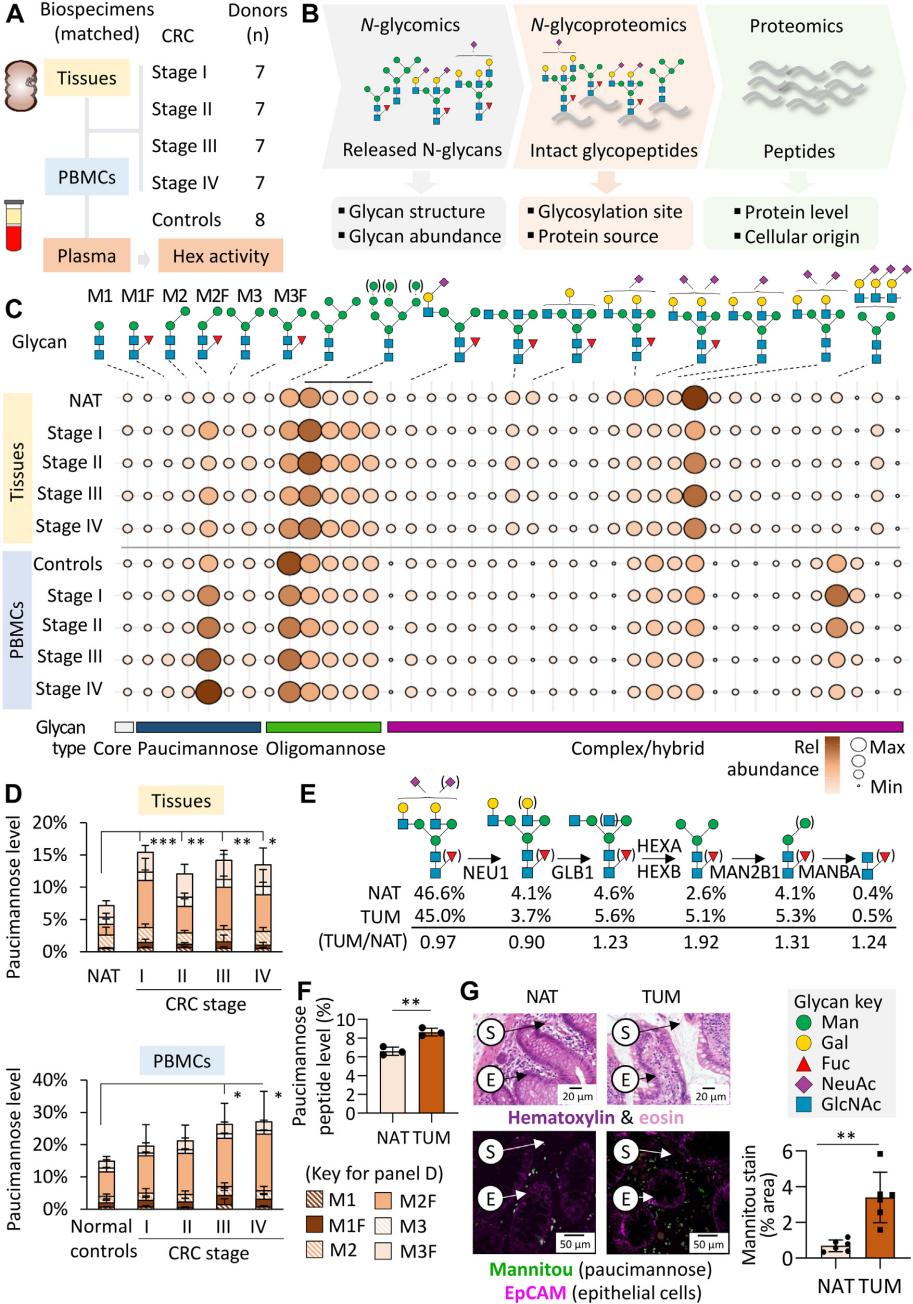
**Strong paucimannosidic glyco-signatures in CRC tumor tissues and PBMCs.***A*, sample cohort used for the discovery phase of the study including resected tumor tissues (TUMs), PBMCs, and plasma from 28 patients with CRC (stage I–IV). Normal adjacent tissues (NATs) from 8 CRC patients (stage I) and paired PBMCs and plasma from eight non-CRC (normal) donors were used as controls (see Supplemental Table S1 for details). *B*, systems glycobiology was used to map the expression and to suggest cellular origin(s) of glycoproteins across the CRC stages (relative to controls) in tumor tissues and PBMC samples. *C*, comparative glycomics of matched tumor and PBMC samples across the CRC stages compared to controls (see Supplemental Tables S4 and S5 for data). M1-M3F: Paucimannosidic *N*-glycans. *D*, stage-specific expression levels of protein paucimannosylation in CRC tumors (*upper panel*) and PBMCs (*lower panel*) relative to controls as measured by quantitative glycomics (n = 7/CRC stage, n = 8 controls, student’s *t* test, ∗*p* < 0.05, ∗∗*p* < 0.01, ∗∗∗*p* < 0.001). *E*, expression levels of *N*-glycans within the truncation pathway and their biosynthetic precursors as determined from quantitative glycomics of FFPE sections of TUM and NAT from a CRC patient (stage II) (see Supplemental Table S6 for data). Truncation pathway details were from (44, 45). *F*, Paucimannosidic peptide levels based on glycoproteomics of FFPE sections of TUM *versus* NAT from CRC patients (stage II) (n = 3, student’s *t* test, ∗∗*p* < 0.01, see Supplemental Table S7 for data). *G*, representative examples of tissue architecture (H&E stain, *magenta* and *pink*) and paucimannose and EpCAM expression as determined with a paucimannose-reactive antibody (Mannitou, *green*) and an epithelial cell-specific antibody (anti-EpCAM, *purple*) in paired TUM and NAT from a CRC patient (stage II). Mannitou staining was quantified from six different images (Supplemental Fig. S1). E = epithelium, S = stroma region. CRC, colorectal cancer; EpCAM, epithelial cellular adhesion molecule; FFPE, formalin-fixed paraffin-embedded; NAT, normal adjacent tissue; PBMC, peripheral blood mononuclear cell; TUM, tumor tissues.

Aiming to discover new glyco-markers against CRC, this study first employed a multi-faceted untargeted -omics approach to map glycoproteome alterations associated with CRC across various biospecimens (snap-frozen and FFPE TUMs, PBMCs) from CRC patients and matching controls (see Supplemental Table S1-S3 for overview of samples and experimental approaches). Systems glycobiology—namely, integrated glycomics, glycoproteomics, and proteomics—was used to globally quantify *N*-glycan structures and their sites and protein carriers, as well as provide clues to their cellular origins from snap-frozen tissues and PBMCs of 28 CRC patients (n = 7/stage) (Fig. 1, *A* and *B*). In addition to the resected CRC TUMs, NATs were available from CRC patients in disease stage I (n = 8), whereas PBMCs from healthy donors (n = 8) were used as controls in those experiments.

Unlike the conventional *N*-glycan types (oligomannosidic-, hybrid-, complex-type), the less investigated non-canonical paucimannosidic-type glycans, that is, Man_1-3_GlcNAc_2_Fuc_0-1_ (short-hand M1-M3F) (42, 43, 44) were strongly elevated in the *N*-glycome of CRC TUM (CRC stage I-IV: 13.5–15.4%, NAT: 6.7%) and in PBMCs (CRC stage I–IV: 19.5–27.1%, normal controls: 14.9%) as determined using quantitative glycomics (Fig. 1*C*, see Supplemental Tables S4 and S5 for data and Supplemental Data S1 for spectral evidence). Significant elevation in protein paucimannosylation in the CRC TUM was observed across all disease stages compared to NAT and in PBMCs from CRC patients with advanced disease (stage III–IV) relative to PBMCs from normal controls (Fig. 1*D*). The core fucosylated M2F and M3F structures (Man_2-3_GlcNAc_2_Fuc_1_) dominated in both CRC TUM and PBMCs covering 60 to 70% of the paucimannosidic *N*-glycans. While still largely unexplored in cancer cells, the formation of paucimannosidic *N*-glycans through an only recently discovered truncation pathway involves most critically the HEXA and HEXB among other glycoside hydrolases to form M3(F) and further truncated structures (Fig. 1*E*) (44, 45). Recapitulating findings from the snap-frozen CRC tissues, quantitative glycomics of FFPE sections from colonic tissue of a CRC patient (stage II) showed that paucimannosidic *N*-glycans are elevated in CRC TUM compared to NAT (TUM/NAT ratio > 1), while the elongated *N*-glycans (paucimannose precursors) showed the opposite pattern (lower TUM/NAT ratio) (see Supplemental Table S6 for data). Quantitative glycoproteomics of FFPE sections confirmed the elevation of paucimannosidic peptides in TUM over NAT (Fig. 1*F*, see Supplemental Table S7 for data). Using IHC, we also demonstrated an increased staining response against Mannitou, a paucimannose-reactive antibody (32, 33), in FFPE TUM sections from a CRC patient (stage II) compared to matching NAT (Fig. 1*G* and Supplemental Fig. S1).

### Paucimannosidic Proteins of Likely Immune and Cancer Cell Origin Dominate in CRC Tumors

**Figure 2:**
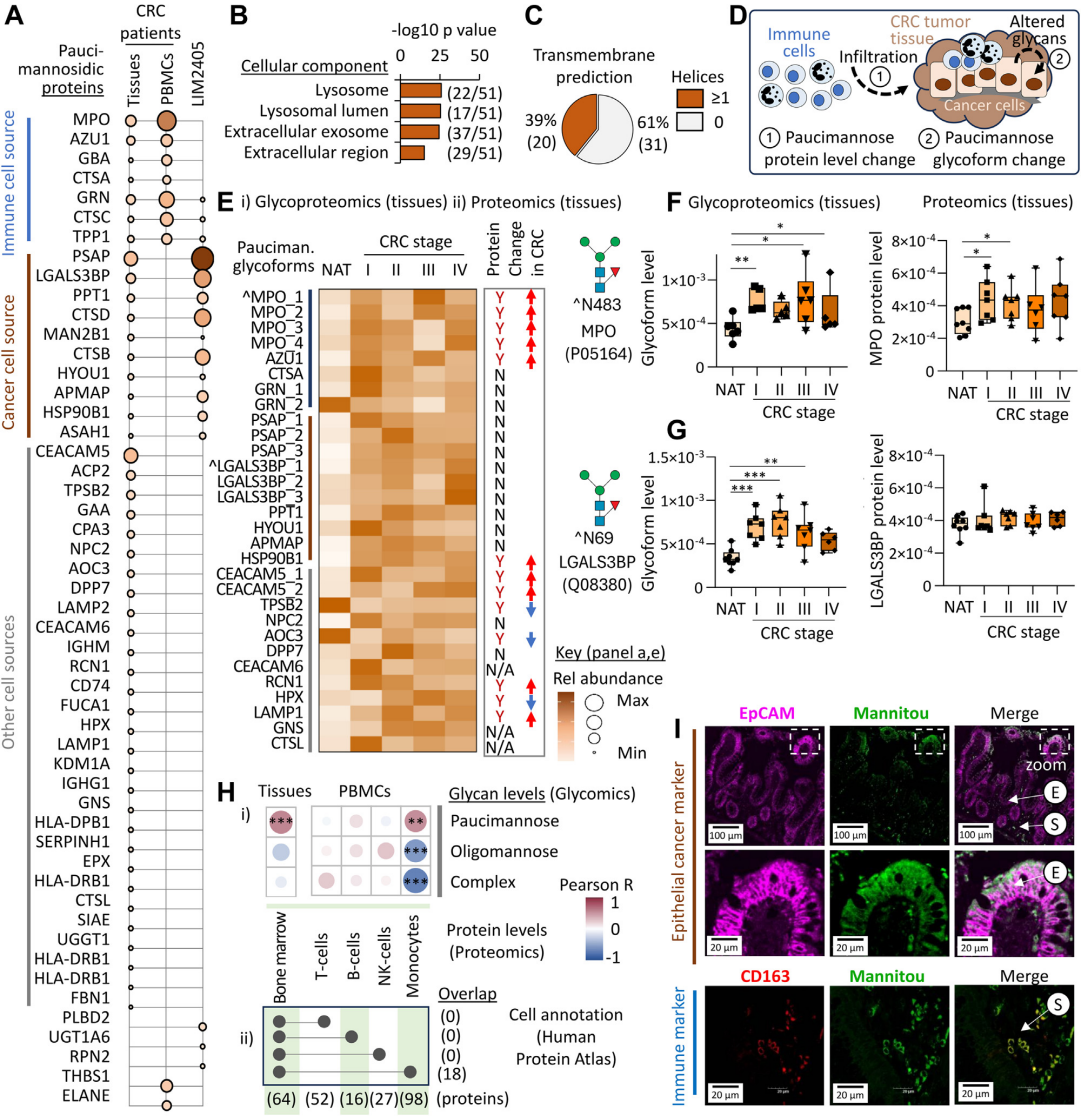
**Paucimannosidic proteins of likely monocytic and cancer cell origins are prominent features in CRC tumor tissues.***A*, expression level of paucimannosidic proteins identified in CRC tumor tissues, PBMCs and in a CRC patient–derived cell line (LIM2405) using glycoproteomics (see Supplemental Tables S8–S10 for data and panel E for key). Prediction of (*B*) cellular localization (gene ontology) and (*C*) transmembrane regions (TMHMM 2.0) of the paucimannosidic proteins identified in CRC tumor tissues, PBMCs and LIM2405 cells. *D*, schematics of two putative mechanisms (labeled as 1 and 2) contributing to elevated paucimannosidic signatures in CRC tumor tissues. *E*, overview of (i) site-specific paucimannosidic glycoform distribution measured by glycoproteomics and (ii) protein levels measured by proteomics of snap frozen CRC TUM and NAT (see Supplemental Tables S8 and S11 for data). Each row represents a unique paucimannosidic glycoform (unique glycosylation site and paucimannosidic structure). Only paucimannosidic glycoforms showing significant differences are listed (n = 7/stage, n = 8 controls, student’s *t* tests, *p* < 0.05, N/A = protein not detected in dataset). *Arrows*: direction of change in CRC TUM *versus* NAT. ˆSpecific paucimannosidic glycoforms visualized in panel *F*-*G*. Expression of a paucimannosidic M3F glycoforms (*left*) and/or protein level (*right*) in CRC TUM *versus* NAT for (*F*), MPO (N483) of putative immune cell origin and (*G*) LGALS3BP (N69) of putative CRC cell origin (n = 7/CRC stage, n = 8 controls, student’s *t* test, ∗*p* < 0.05, ∗∗*p* < 0.01, and ∗∗∗*p* < 0.001). *H*, i, correlation analysis of cell-specific protein markers (proteomics) and glycosylation type (glycomics) in snap-frozen CRC tissues and PBMCs. Pearson correlation (n = 36), ∗*p* < 0.05, ∗∗*p* < 0.01, and ∗∗∗*p* < 0.001. ii, proteins identified in the snap-frozen CRC tissues and PBMCs were annotated for putative cellular origin using the Human Protein Atlas and their overlap plotted. *I*, IHC analysis targeting paucimannosidic glycoepitopes (Mannitou, *green*), epithelial cancer cells (anti-EpCAM, *magenta*) and anti-inflammatory macrophages arising from tumor-infiltrating monocytes (anti-CD163, *red*) in different slides (z stacks) from the same FFPE sections of CRC TUM from a CRC patient (stage II). Colocalization (*yellow*) was visually assessed (*merge*). E = epithelium, S = stroma. CRC, colorectal cancer; EpCAM, epithelial cellular adhesion molecule; FFPE, formalin-fixed paraffin-embedded; IHC, immunohistochemistry; NAT, normal adjacent tissue; PBMC, peripheral blood mononuclear cell; TUM, tumor tissue.

Glycoproteomics identified 51 paucimannosidic proteins in the CRC TUMs (Fig. 2*A*, see Supplemental Table S8 for data). Glycoproteomics of matching PBMC samples from CRC patients and a patient-derived CRC cell line (LIM2405) identified 7 (13.7%) and 13 (25.5%) of these 51 paucimannosidic proteins, respectively, suggesting immune and CRC cell origins of a subset of the paucimannosylated proteins in CRC tumors (see Supplemental Tables S9 and S10 for data). Cellular component analysis showed that the paucimannosidic proteins identified in the CRC TUMs were mainly from lysosomal and extracellular regions (Fig. 2*B*) and spanned both soluble and membrane-tethered proteins (Fig. 2*C*).

Building on our reports that circulating innate immune cells (*e*.*g*., neutrophils, monocytes) express and store paucimannosidic proteins in intracellular lysosomal (or lysosomal-like) compartments that may be released upon cell activation (42, 46, 47, 48, 49, 50) and the established knowledge that innate immune cells abundantly infiltrate the CRC tumor microenvironment (TME) (51, 52), these observations led us to hypothesize that the strong paucimannosidic signatures in CRC TUMs may, in part, originate from infiltrating immune cells and, in part, arise from aberrations in the glycosylation machinery of the cancerous epithelial CRC cells (Fig. 2*D*).

Supporting our hypothesis, we showed that for key innate immune-derived proteins (*e*.*g*., MPO, AZU1), the elevation in paucimannosidic glycoforms was generally accompanied by an elevation in their respective protein levels in CRC TUM relative to NAT (Fig. 2*E*, see Supplemental Tables S8 and S11 for data). Site-specific glycoprofiling of MPO substantiated this relationship as illustrated for an M3F-peptide spanning the Asn483 site (Fig. 2*F*). In contrast, for proteins putatively annotated to arise from CRC cells (*e*.*g*., PSAP, LGALS3BP), the elevation in paucimannosidic glycoforms were generally not accompanied by changes in their protein levels, as exemplified by the site-specific glycoprofiling of a M3F-peptide spanning the Asn69 site of LGALS3BP (Fig. 2*G*).

To detail the immune cell origin(s) of the paucimannosidic signatures in the CRC TME, we used quantitative proteomics and glycomics and the well-curated HPA (tissue section) as a robust reference for putative cell origin. The expression of the CRC TUM-resident proteins putatively annotated to arise from immune cell populations (bone marrow origin) displayed a strong association with paucimannosylation levels (Pearson correlation, R = 0.57, *p* = 0.0003, n = 36) and not with oligomannosidic- and complex-type glycosylation (Fig. 2Hi, see Supplemental Tables S4 and S11 for data). Supporting this relationship, quantitative proteomics of FFPE sections of colonic tissues from a CRC patient (stage II) revealed increased levels of immune cell–annotated proteins, some of which were found carry paucimannosylation, in CRC TUM relative to NAT (Supplemental Fig. S2, see Supplemental Table S13 for data). Additionally, paucimannose levels correlated positively (Pearson R = 0.52, *p* = 0.001, n = 36) and oligomannosidic- and complex-type glycosylation correlated negatively with the expression of CRC PBMC proteins that were putatively annotated to arise from monocytes (and not from cells of lymphocytic origin, *i*.*e*., T cells, B cells, and NK cells) using blood section of the HPA (see Supplemental Tables S5 and S12 for data). Notably, only proteins of monocytic origin (not from T cells, B cells, and NK cells) were found among the immune cell–annotated proteins identified in the CRC TUMs, indicating that the paucimannosidic signatures in the CRC TME, at least in part, originate from tumor-infiltrating monocytes (Fig. 2*H*, ii).

IHC of FFPE sections of CRC TUM (stage II) revealed that while little direct co-localization between Mannitou (paucimannosylation) and EpCAM (epithelial cells) was observed, considerable Mannitou staining was observed peripheral to the epithelium (Fig. 2*I*). Supported by the -omics data, these observations suggest that epithelial cancer cells release lysosomal paucimannosidic proteins (*e*.*g*., PSAP) into the CRC TME consistent with similar observations from another study (53). Importantly, paucimannosidic glycoepitopes were found to colocalize directly with CD163+ anti-inflammatory macrophages in the CRC TUMs. Our findings therefore support that both tumor-infiltrating monocytes (ultimately macrophages) and epithelial cancer cells contribute to the strong paucimannosidic signatures found in CRC TUMs.

### HEXB is Elevated in CRC and Drives Paucimannosidic Protein Formation in CRC Cells

**Figure 3:**
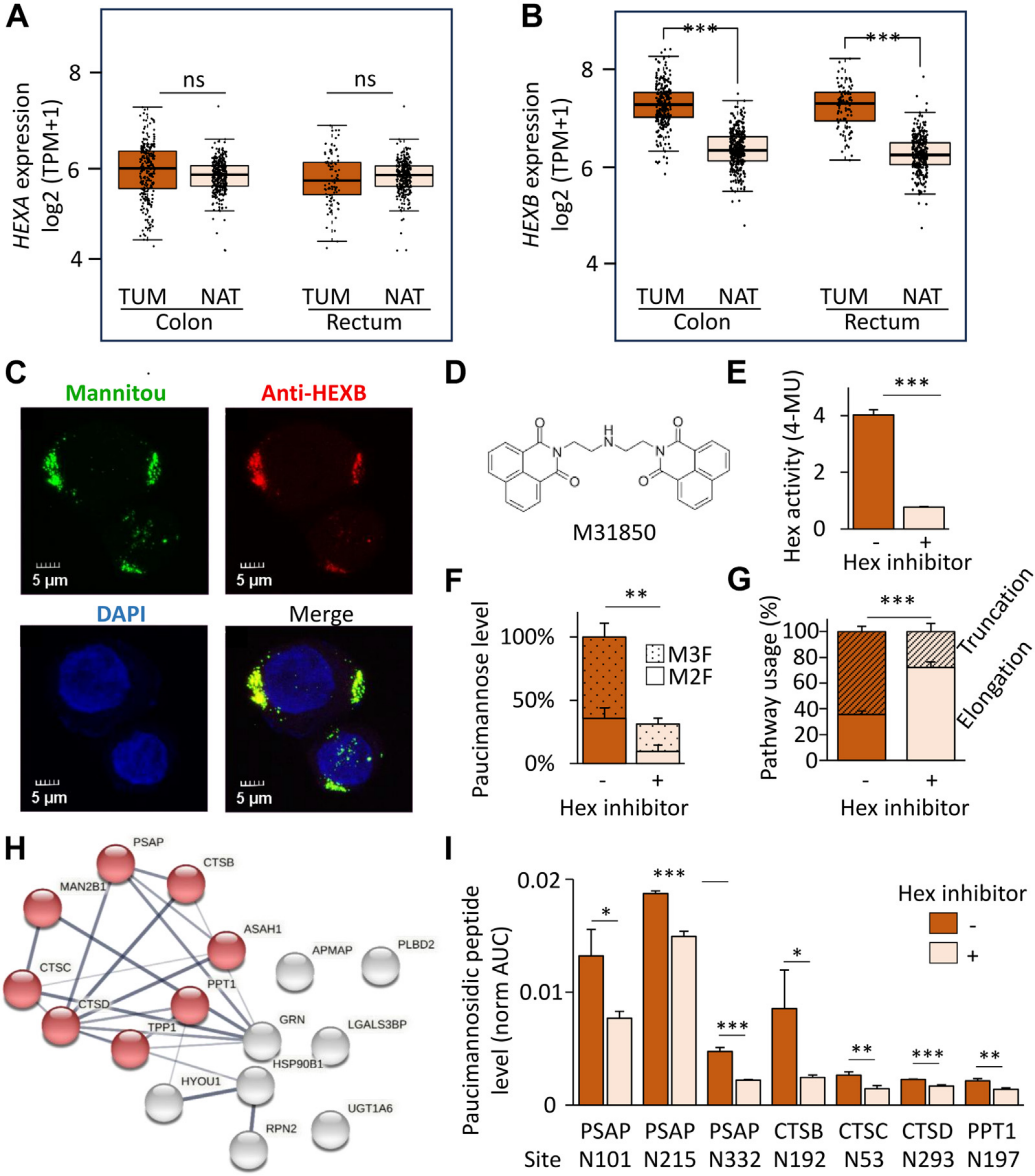
**HEXB drives paucimannosidic protein formation in CRC cells.** Gene expression (mRNA levels) of (*A*) *HEXA* and (*B*) *HEXB* in colon cancer (n = 275) *versus* NAT (n = 349) and rectal cancer (n = 92) and NAT (n = 318) tissues cohorts. Data from TCGA and GTEx databases. ∗∗∗Adjusted *p* < 0.001. *C*, immunocytochemistry of LIM2405 cells using paucimannose-reactive Mannitou (*green*) and anti-HEXB (*red*) antibodies. 4′,6-diamidino-2-phenylindole (DAPI) (*blue*). *D*, chemical structure of M31850, a known pharmacological Hex inhibitor (55). *E*, total Hex activity of LIM2405 cells with and without Hex inhibition as measured using an established MUG substrate assay. *F*, relative levels of fucosylated paucimannose (M2F, M3F, Fig. 1*C* for structures) in LIM2405 cells with and without Hex inhibition. *G*, *N*-glycome changes in LIM2405 cells upon Hex inhibition assessed by the relative pathway usage. For panel E-G: Hex inhibited LIM2405 cells were compared to vehicle controls using student’s *t*-tests (n = 3 technical replicates per experiment, ∗∗*p* < 0.01, ∗∗∗*p* < 0.001, see Supplemental Table S14 for data). *H*, network analysis of the paucimannosidic proteins identified in LIM2405 using STRING software (https://string-db.org/). Nodes highlighted in *red* are involved in lysosomal pathways/activity. *I*, site-specific paucimannose levels in a subset of the identified paucimannosidic proteins in Hex inhibited and untreated LIM2405 cells as measured by quantitative glycoproteomics (n = 3 technical replicates per experiment, ∗*p* < 0.05, ∗∗*p* < 0.01, and ∗∗∗*p* < 0.001, see Supplemental Table S10 for data). CRC, colorectal cancer; GTEx, Genotype-Tissue Expression; HEXA, *N*-acetyl-β-D-hexosaminidase subunit α; HEXB, *N*-acetyl-β-D-hexosaminidase subunit β; MUG, 4-methylumbelliferyl-2-acetamido-2-deoxy-β-D-glucopyranoside; TCGA, The Cancer Genome Atlas; TPM, transcripts per million.

Guided by our -omics-based observations of elevated paucimannosylation in CRC tumors, we turned our attention to HEXA and HEXB, two hydrolytic lysosomal isoenzymes known to catalyze the formation of paucimannosidic proteins in human neutrophils (45). Quantitative proteomics found that HEXB (rather than HEXA) dominates in CRC TUMs and PBMCs (Supplemental Fig. S3, *A* and *B*). Gene expression (transcript) data of large cohorts of colon and rectum TUM and paired NAT samples from The Cancer Genome Atlas and Genotype-Tissue Expression showed that while the less expressed *HEXA* remained unchanged between TUM and NAT (Fig. 3*A*), the highly expressed *HEXB* was elevated in both colon (*p* < 0.001, n = 275–349) and rectum (*p* < 0.001, n = 92–318) TUM relative to NAT (Fig. 3*B*). Surprisingly, a HEXB-focused reinterrogation of quantitative proteomics data of paired tissue samples from 96 CRC patients available from Clinical Proteomic Tumor Analysis Consortium (35) showed only mild (insignificant) increase of HEXB protein levels in TUM compared to NAT (Supplemental Fig. S4*A*). While disparate mRNA><protein relationships have been reported by others (54) and at this point cannot be ruled out to explain our observation, an alternative explanation may be that HEXB after expression within the TME by tumor-infiltrating monocytes and/or epithelial CRC cells (both of which actively express *HEXB*, Supplemental Fig. S4, *B* and *C*), leaves the TUMs to enter circulation. Supporting tumor secretion of HEXB, reinterrogation of quantitative proteomics data of a large CRC plasma cohort (36), revealed elevated HEXB protein levels in plasma from CRC patients relative to healthy controls (Supplemental Fig. S4*D*). Further indicating the involvement of HEXB in the formation of paucimannosidic proteins in CRC cells, HEXB accurately colocalized with paucimannosidic glycoepitopes in LIM2405 cells as assessed by immunocytochemistry (Fig. 3*C*).

To validate that HEXB catalyzes paucimannosidic protein formation in CRC cells, we used a pharmacological Hex inhibitor (M31850) (55) (Fig. 3*D*) in LIM2405 cells with the aim to block the first step of the truncation pathway in which paucimannosidic proteins are formed (Fig. 1*E*). We first tested across an extended concentration range the impact of M31850 and vehicle (DMSO) on the viability of LIM2405 cells (Supplemental Fig. S5*A*). At 31.25 μM M31850, the Hex enzyme activity was effectively inhibited without compromising the cell viability caused by higher vehicle concentrations (Fig. 3*E* and Supplemental Fig. S5*B*).

Glycomics profiling showed an M31850 dose-dependent decrease in the levels of paucimannosylation relative to the vehicle control confirming that the Hex enzyme (predominantly HEXB) catalyzes paucimannosidic protein formation in CRC cells (Supplemental Fig. S5*C*). At 31.25 μM M31850, the LIM2405 cells displayed a significant reduction in protein paucimannosylation mainly driven by the fucosylated paucimannosidic glycans (M2F and M3F) (Fig. 3*F*, see Supplemental Table S14 for data) that were found to be particularly prominent in CRC TUMs (Fig. 1*D*). As expected, Hex inhibition led to a considerable switch in the biosynthetic *N*-glycosylation pathways now favoring elongation over truncation (Fig. 3*G*). Supporting these findings, glycoproteomics data showed an M31850-induced decrease of several paucimannosidic glycan structures across 31 glycosylation sites covering 16 proteins (Supplemental Table S10). In line with observations made for the CRC TUMs (Fig. 2*B*), the paucimannosidic proteins identified in the LIM2405 cells were predominantly of lysosomal origin (KEGG pathway, Benjamini FDR = 2.0^−9^), including ASAH1, CTSB, CTSC, CTSD, MAN2B1, PPT1, PSAP, and TPP1 (Fig. 3*H*), five of which showed an M31850-induced reduction in paucimannosylation across seven sites (Fig. 3*I*).

### Total Plasma Hex Activity Stratifies CRC Patient Risk

Given the biosynthetic relationship between elevated HEXB and raised paucimannosylation in CRC, we sought to investigate the prognostic value of using the plasma Hex activity to report on CRC patient risk in terms of their 5-year survival outcome. As routinely performed to diagnose Hex deficiency in Tay-Sachs (*HEXA*^−/−^) and Sandhoff (*HEXB*^−/−^) disease (56), Hex activity can be readily measured in blood plasma using fluorescent substrates, that is, MUG (measuring total Hex activity) and MUGS (measuring HEXA-specific activity). In line with the observation that HEXB dominates in CRC TUMs and PBMCs (Supplemental Fig. S3, *A* and *B*, and Fig. 3, *A* and *B*), the activity of HEXB (measured as MUG-MUGS) was found to be the principal Hex isoenzyme form in CRC plasma (Supplemental Fig. S3*C*). Given the insignificant activity of HEXA, the downstream experiments were limited to measuring total Hex activity (MUG) effectively serving as a proxy for the HEXB-specific activity.

**Figure 4:**
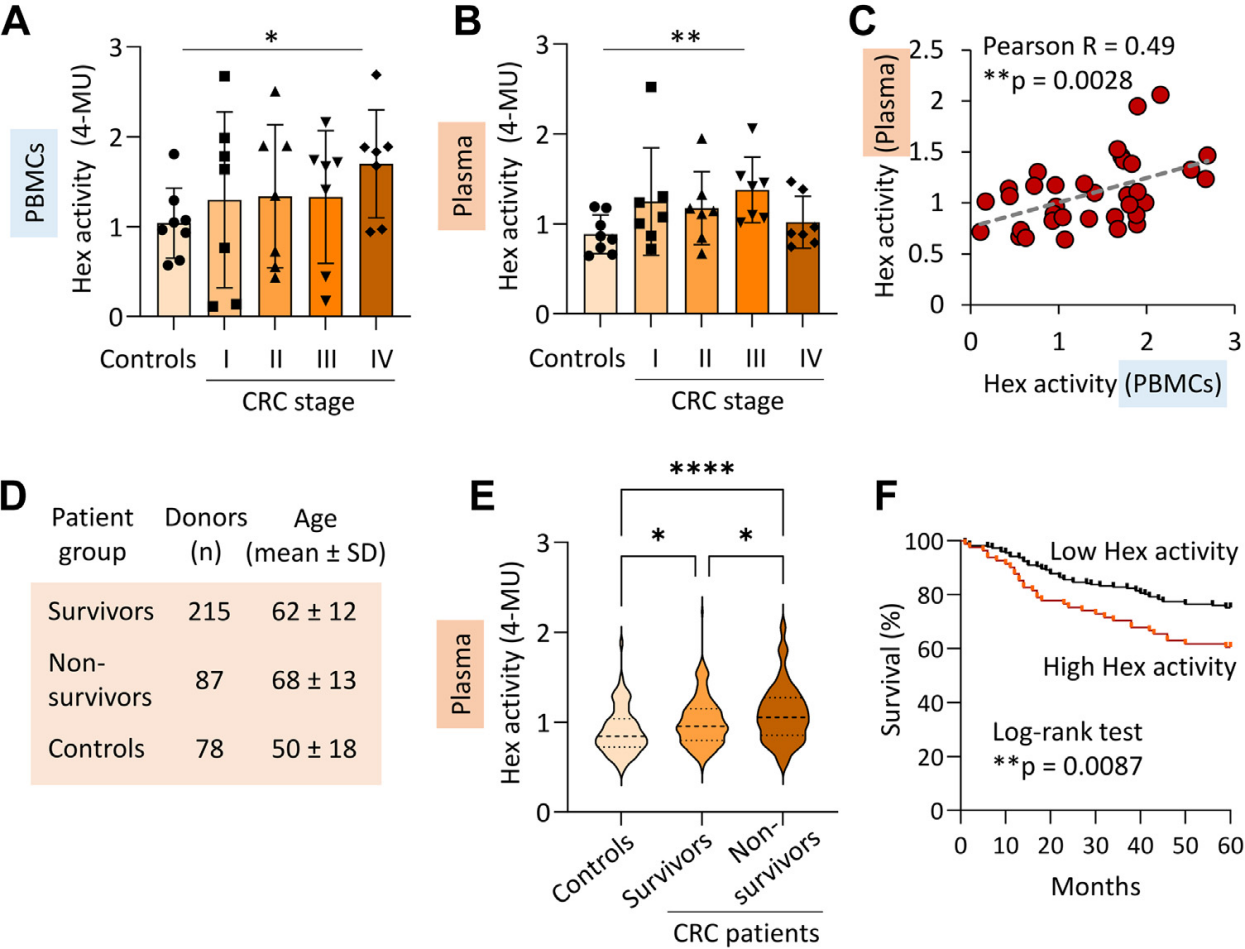
**Plasma Hex activity associates with CRC mortality enabling patient risk stratification.** Elevation of total Hex activity in matched (*A*) PBMCs and (*B*) plasma samples in CRC patients with advanced disease relative to healthy controls as measured using an established MUG assay (n = 7/stage, n = 8 controls, student’s *t*-tests, ∗*p* < 0.05, ∗∗*p* < 0.01). *C*, correlation between total Hex activity in the paired PBMC and plasma samples from the same individuals (Pearson, 95% confidence interval, n = 35. One outlier was identified in plasma using the ROUT method and was removed). *D*, plasma from a large clinical cohort including both CRC survivors (n = 215) and nonsurvivors (n = 87) (determined 5 years postsurgery/treatment) and control non-CRC (normal) donors (n = 78) were profiled for total Hex activity. *E*, plasma Hex activity. See Supplemental Table S3 for data. ANOVA followed by Tukey multiple comparison (∗∗∗∗ adjusted *p* < 0.0001, ∗adjusted *p* < 0.05). *F*, CRC patients with high plasma Hex activity (*red trace*) exhibited lower 5-year survival relative to CRC patients with lower plasma Hex activity (*black trace*) (log-rank test, *p* = 0.0087). Boundary for high/low Hex activity was determined by 75th percentile (high, n = 81, low, n = 221). CRC, colorectal cancer; Hex, N-acetyl-β-D-hexosaminidase; MUG, 4-methylumbelliferyl-2-acetamido-2-deoxy-β-D-glucopyranoside; PBMC, peripheral blood mononuclear cell.

Consistent with the elevated HEXB protein levels in CRC plasma (Supplemental Fig. S4*D*), the Hex enzyme activity appeared mildly elevated in PBMCs and plasma from CRC patients with late-stage disease (stage III–IV) compared to controls (Fig. 4, *A* and *B*). Paired analysis serving to remove the considerable inter-individual variation in this small patient cohort, correlations (Pearson R = 0.49, *p* = 0.0028) were observed between the total Hex activity in plasma and PBMCs across the 35 matched sample sets, suggesting that circulating HEXB, at least in part, arises from PBMCs (Fig. 4*C*). Supporting this hypothesis, *HEXB* is known to be actively expressed in all immune cells forming the PBMC fraction and is particularly highly expressed in monocytes (normalized transcripts per million = 343.7) (Supplemental Fig. S4*B*). In fact, the monocytic expression of *HEXB* was estimated to be 2.5 higher than in CRC cells (average normalized transcripts per million = 133.0 determined across 63 cell lines) (Supplemental Fig. S4*C*). Taken together, our collection of -omics data suggests that tumor-infiltrating monocytes contributes to the enhanced *HEXB* gene expression in CRC tumors, which results in elevated copy numbers of soluble HEXB protein molecules that are predominantly secreted into circulation. However, further studies, for example, using quantitative proteomics and activity assays of various single cell populations isolated from resected tumors are required to establish the exact contribution of HEXB from infiltrating monocytes (and other immune populations) and epithelial CRC cells to the raised levels observed in circulation in CRC patients. Adding complexity to such investigations, the cellular content and complexity of the TME appeared to vary significantly between patients (Supplemental Table S1) suggesting different immune cell infiltration rates and involvement in CRC and likely explaining the relatively high interpatient variation in molecular features reported in this study (*e*.*g*., Figs. 1*D*, 2, *F* and *G*, and 4, *A* and *B*).

Importantly, the plasma Hex activity appeared both biologically stable as measured across repeat drawings from the same donor (Supplemental Fig. S6*A*) and technically stable as shown across multiple freeze/thaw cycles and storage conditions (Supplemental Fig. S6, *B* and *C*). Additionally, the Hex activity assay showed robustness and reproducibility across different blood sample types, including serum, EDTA- or heparin-plasma (Supplemental Fig. S6*D*). Collectively, these favorable characteristics indicate that the plasma Hex activity assay is compatible with use in a clinical setting.

To investigate the potential of the plasma Hex activity to stratify CRC patient risk in terms of mortality, total Hex activity was measured in plasma from a large cohort of 380 plasma samples comprising CRC patients for which 5-year survival data were available and healthy donors (Fig. 4*D*). The cohort included CRC patients who survived (n = 215) and patients who did not survive (n = 87) the disease 5 years postsurgery/treatment. Notably, the plasma Hex activity was significantly higher in nonsurviving CRC patients than both the CRC survivors (adjusted *p* = 0.0372, area-under-the-curve (AUC) = 0.581) and heathy controls (adjusted *p* < 0.0001, AUC = 0.685) as well as in CRC survivors compared with healthy controls (adjusted *p* = 0.016, AUC = 0.685) (Fig. 4*E*). Accordingly, we found that CRC patients featuring high plasma Hex activity (1.39 ± 0.21 relative Hex activity units, n = 81) experienced a significantly higher 5-year mortality risk than patients with low plasma Hex activity (0.87 ± 0.16 relative Hex activity units, n = 221, log-rank test, *p* = 0.0087) (Fig. 4*F*). Intuitively, mortality is associated with age. Thus, we performed a hazard ratio analysis using a Cox-model to determine the prognostic value of plasma Hex activity when considering age as a confounding factor (Table 1). When adjusted for age, the plasma Hex activity was still significantly associated with mortality; CRC patients had 2.04 times higher risk of mortality over 5 years per unit of plasma Hex activity. Although not assessed in this study, multivariate models factoring in other clinical parameters (*e*.*g*., medication, underlying health conditions) or individual characteristics (*e*.*g*., gender, ethnicity) may improve further the use of plasma Hex activity to predict survival of CRC patients. This study has shown that the plasma Hex activity, which can be easily measured using a fast, sensitive, and robust assay, may have clinical utility for prognostication of CRC patient risk.

**Table 1:** Hazard ratio to assess impact of age and plasma Hex activity on 5-year CRC patient survival

	Mean (SD)	HR (univariable)	HR (multivariable)
Age (years)	63.9 (12.7)	1.03 (1.01–1.05) *p* = 0.001	1.03 (1.01–1.05) *p* = 0.004
Hex activity (rel. activity units)	1.0 (0.3)	2.49 (1.27–4.86) *p* = 0.008	2.04 (1.01–4.11) *p* = 0.046

CRC, colorectal cancer; Hex, N-acetyl-β-D-hexosaminidase; HR, hazard ratio.

## Discussion

CRC remains a life-threatening disease featuring high morbidity and mortality (1). With the goal of identifying new easy-to-assay molecular markers for CRC to inform the clinical decision-making and improve patient outcomes, we have here applied untargeted systems glycobiology approaches to biospecimens from CRC patients revealing that noncanonical paucimannosidic proteins and the underpinning biosynthetic glyco-enzyme, HEXB, are molecular signatures that accompany CRC and that the plasma Hex activity is a promising prognostic marker candidate to stratify CRC patient survival.

While protein paucimannosylation is a constitutively expressed and tissue-wide *N*-glycan modification in plants and lower vertebrates and until recently considered absent in mammals (57, 58), our developing knowledge of paucimannose in human glycobiology points to a tissue-restricted and context-dependent expression of this noncanonical class of glycoproteins in humans (44) with reports mainly related to immunity (42, 46, 47, 48, 49, 59) and cancer (21, 60, 61, 62, 63) including in CRC (14, 64, 65, 66). These recent observations of noncanonical paucimannosylation in cancerous biospecimens (including those reported in this study) were enabled by technology improvements in the emerging field of systems glycobiology (67). Burgeoning glycomics and glycoproteomics methods are uniquely able to comprehensively survey the glycoproteome at scale even in complex tissues and cellular environments and accurately report on quantitative changes of glycosylation features including unusual (unexpected) structures previously overlooked with targeted analytical methods. The fact that paucimannosylation in this study was among the glycan types displaying the greatest elevation in both CRC TUMs and PBMCs relative to matched controls made this initial -omics-based observation a particularly interesting lead to follow.

Identifying the cell source(s) of proteins let alone their glycan modifications in complex biological samples such as the investigated CRC TUMs or PBMC samples comprising diverse cell populations remains a considerable challenge in glycobiology. Enabled by an innovative correlation approach that leveraged our quantitative -omics data of multiple CRC specimens, including tissue, PBMCs, and a well-established CRC cell line model (68), and annotation of proteins to their putative cell- and tissue origins by the well-curated HPA (24), our data suggest that the paucimannosidic signatures in CRC TUMs arise from multiple cellular sources including from tumor-infiltrating monocytes and epithelial cancer cells present in the CRC TME. Supporting these findings, we and others have found paucimannosylation to be an abundant glycan feature in isolated blood monocytes and in macrophage effector cells derived from monocytes (47, 69, 70) as well as in various CRC cell lines (15, 65). We have also recently shown that cancerous epithelial cells frequently modify their lysosomal proteins with paucimannosidic *N*-glycans and have mechanistically detailed how the lysosomal content in such cells may be released to the extracellular environment *via* regulated exocytosis through focal adhesion points (53). Release of paucimannosidic proteins from epithelial cancer cells through extracellular vesicles was also reported (71). However, the sheer abundance of paucimannose in blood monocytes (∼30% of the monocytic *N*-glycome) (47) and the significant infiltration rate of monocytes into the CRC TME (72), point to the tumor-associated myeloid cells being principal contributors to the prominent paucimannosidic features found in CRC TUMs. Consistently, our IHC analysis demonstrated prominent colocalization of paucimannosidic epitopes with anti-inflammatory (CD163+) macrophages known to originate from tumor-infiltrating monocytes (Fig. 2*I*). The observation of paucimannose-rich anti-inflammatory macrophages is particularly interesting as this macrophage subpopulation is known to support tumor growth and spread (73); however, this study did not explore any functional links between paucimannose (and HEXB) and CRC, which therefore remains to be investigated. Because PBMCs and TUMs are cellularly heterogenous specimens, we cannot rule out that other cellular sources including proinflammatory macrophages and other cells in the CRC TME (*e*.*g*., tumor-infiltrating neutrophils, dendritic cells, T cells, NK cells, B cells, and fibroblasts) may also contribute to the paucimannosidic signatures in the CRC TUMs. Such details may now be explored using new spatial -omics approaches (*e*.*g*., MALDI-MS imaging), advanced microscopy (*e*.*g*., FRET) or cell sorting followed by single cell population glyco(proteo)mics analysis (74, 75, 76).

We have previously shown that paucimannosidic proteins in human neutrophils are biosynthesized by the Hex isoenzymes (45), but the enzyme(s) responsible for raised paucimannose levels in CRC have so far remained unknown despite previous associations pointing to the Hex isoenzymes also being responsible in cancer cells (63). As *HEXB* (not *HEXA*) expression in this present study was found to be elevated in CRC tissues from a large patient cohort and the fact that the HEXA protein level appeared insignificant in the investigated CRC specimens (Fig. 3, *A* and *B*, and Supplemental Fig. S3, *A* and *B*), we focused the downstream investigation on HEXB. Using IHC and a Hex inhibitor (M31850) on a patient-derived CRC line (LIM2405), we documented the biosynthetic link between HEXB and paucimannosidic proteins in CRC cells. In parallel efforts, we have recently used *HEXA* and *HEXB* KO lines to demonstrate the existence of the noncanonical truncation pathway in other cancer cells indicating that Hex-mediated paucimannosidic protein formation may be a biosynthetic pathway generally active across various cancer cell types (thus not unique to CRC) (53). Although not assessed in this study, HEXB can also act on other glycoconjugates, such as glycolipids, *O*-glycoproteins and glycosaminoglycans (77, 78). Considering these multiple substrate activities becomes important for future studies setting out to mechanistically delineate functional roles of HEXB in CRC progression. Moreover, while parallels to the human neutrophils are tempting to draw given their common progenitor origins, the involvement of HEXA and HEXB in paucimannosidic protein formation in human monocytes also awaits exploration.

Aberrations in the expression of paucimannosidic proteins and HEXB in CRC are also interesting in a cancer marker context. However, targeting paucimannosidic proteins and HEXB in blood plasma, the most common bodily fluid for noninvasive biomarkers, appear unviable given that i) glyco(proteo)mics did not identify any paucimannosidic proteins/glycans in CRC plasma (data not shown) possibly due to the dominance of serum glycoproteins carrying complex-type *N*-glycans or the rapid removal of paucimannosidic proteins from circulation (48) and ii) our TMT-based proteomics experiments did not confidently identify and quantify HEXB in nondepleted CRC plasma calling for ultrasensitive proteomics approaches to obtain reliable and consistent HEXB readings in such complex samples (Supplemental Fig. S4*D*). In addition, glycomics and proteomics remain laborious, challenging and costly technologies and therefore largely restricted to specialized laboratories, precluding, at this stage, LC-MS/MS–based measurements of paucimannose and HEXB in the clinic. In contrast, the Hex enzyme activity was readily detected from neat plasma (2 μl/sample) using a simple, sensitive, and robust fluorescent-based substrate assay, which is compatible with clinical routine.

In line with similar findings from other studies reporting that HEXB is overexpressed in CRC cell lines and tissues (20, 61, 79), we found that the Hex enzyme activity is elevated in PMBCs and plasma from CRC patients with advanced stages of the disease relative to matched controls suggesting associations to disease stage and severity. Notably, we also demonstrated that high plasma Hex activity in CRC patients correlates with poor 5-year survival outcomes. The close association between enzyme activity and mortality, suggests that the plasma Hex activity is a promising easy-to-assay marker candidate for disease risk stratification in CRC.

Supporting our findings, serum and urine Hex were previously proposed as putative diagnostic markers for colon cancer (80, 81, 82). However, the fact that several lysosomal glycoside hydrolases including Hex appear raised across a variety of conditions (thus not specific to CRC) limits the potential use of Hex as a diagnostic marker for CRC (83). Given the observed link to patient survival as reported herein, we instead propose that the plasma Hex activity may have prognostic value in terms of disease risk stratification after the CRC diagnosis has been made and preferably at an early stage of the disease where diverse treatment options are available, and interventions are more likely to benefit patients.

In conclusion, we have used new untargeted -omics methods applied to cohorts of patient samples to identify that paucimannosylation are unconventional glycosylation features closely linked to CRC. Prompted by this, we then used a potent enzyme inhibitor to show that HEXB drives paucimannosidic protein formation in CRC cells. Importantly, the Hex enzyme activity was found to be raised in CRC plasma and accurately tracked with patient 5-year survival outcome. Collectively, these findings open new avenues for effective prognostication strategies in CRC.

## Data Availability

The glycoproteomics and proteomics LC-MS/MS raw data have been deposited to the ProteomeXchange Consortium *via* the PRIDE (84) partner repository with the dataset identifiers: PXD051882 (snap-frozen tissue data, username: reviewer_pxd051882@ebi.ac.uk, Password: 6vBY4cxS), PXD051907 (PBMC data, username: reviewer_pxd051907@ebi.ac.uk, Password: 3XDaCtKD), PXD051909 (LIM2405 cell line data, username: reviewer_pxd051909@ebi.ac.uk, Password: vOFYgcR8), and PXD059755 (FFPE tissue data, username: reviewer_pxd059755@ebi.ac.uk, Password: aXCJwmFspnoD. Annotated MS/MS spectral data from proteomics files can be visualized by MSViewer, accession numbers: jl6iaql5wm (snap-frozen tissue data), 9x72xfgesu (PBMC data), zuvgnlr5c3 (LIM2405 data), and hr3mcye83y (FFPE tissue data). Glycomics LC-MS/MS raw data were deposited to GlycoPOST (85) with the identifiers: GPST000423 (snap-frozen tissue data), GPST000424 (PBMC data), GPST000425 (LIM2405 cell line data), and GPST000540 (FFPE tissue data). See Supplemental Table S2 for an overview of raw data files and accession numbers.

## Supporting information

This article contains supporting Information (35, 36).

## Conflict of Interests

The authors declare no competing interests.
