# Supplementary material for: HEXB Drives Raised Paucimannosylation in Colorectal Cancer and Stratifies Patient Risk
Source: Mol Cell Proteomics. 2025 Feb 11;24(3):100927. doi: 10.1016/j.mcpro.2025.100927 (PMC11932691; doi:10.1016/j.mcpro.2025.100927)
Supplement: Supplementary Materials [file mmc2.pdf]

Supplementary Materials

for

HEXB drives raised paucimannosylation in colorectal cancer and stratifies patient risk

Rebeca Kawahara[1,2]*, Liisa Kautto[1], Naaz Bansal[1], Priya Dipta[1], The Huong Chau[1], Benoit Liquet-Weiland[3,4], Seong Beom Ahn[5], and Morten Thaysen-Andersen[1,2]*

[1]School of Natural Sciences, Macquarie University, Sydney, NSW, Australia

[2]Institute for Glyco-core Research (iGCORE), Nagoya University, Aichi, Japan

[3]School of Mathematical and Physical Sciences, Macquarie University, Sydney, NSW, Australia

[4]Université de Pau et Pays de L'Adour, Laboratoire de Mathématiques et de leurs Applications de PAU, CNRS, E2S-UPPA, France

[5]Macquarie Medical School, Faculty of Medicine, Health and Human Sciences, Macquarie University, Sydney, NSW, Australia

***Co-corresponding authors**:

Associate Professor Morten Thaysen-Andersen, PhD (lead contact)
Office 333, Building 4WW, School of Natural Sciences
Macquarie University
NSW-2109, Macquarie Park - Sydney, Australia
Phone: +61 2 9850 7487 / E-mail: morten.andersen@mq.edu.au

Associate Professor Rebeca Kawahara, PhD
Emergent/Innovative Engineering Building Room 815
Nagoya University, Furo-cho, Chikusa-ku, Nagoya, Aichi 464-8601, Japan
Phone:+81 52 558 9724 / E-mail: rebeca.kawahara@igcore.nagoya-u.ac.jp

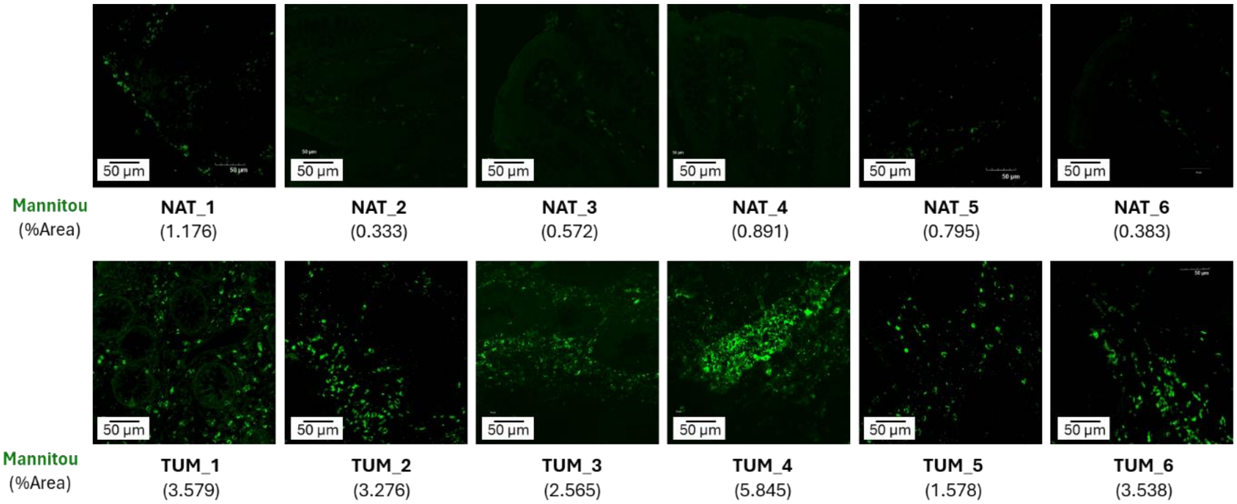

Supplementary Figure S1. IHC analysis of CRC tumor tissues (TUM, lower panels) and normal adjacent tissues (NAT, upper panels) from a CRC patient with stage II disease using paucimannose-reactive antibody (Mannitou, green). Six representative fields were chosen to quantify the Mannitou staining. Scale bar: 50 µm. The staining intensity has been indicated for each field (%Area).

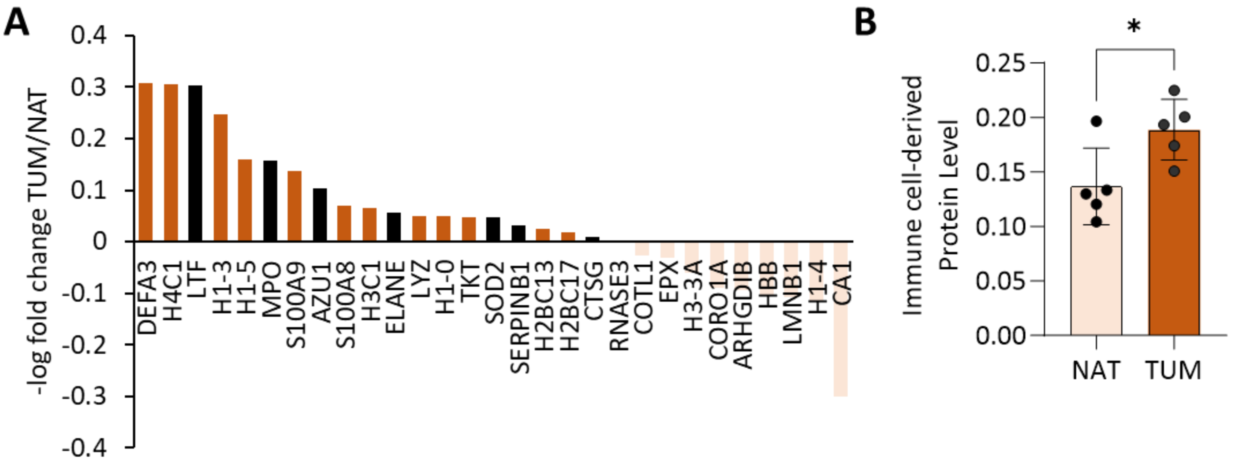

Supplementary Figure S2. Immune cell-derived proteins, some of which carry paucimannosylation, are elevated in CRC tumor tissues. A) Expression differences (average log fold change from five biological replicates, TUM vs NAT) of proteins that were identified in FFPE sections of tumors from a CRC patient (stage II) and suggested to be of immune cell origin based on the Bone Marrow section of the Human Protein Atlas. Black bars denote proteins that were found to carry paucimannosidic glycans. Orange and salmon bars denote non-paucimannosidic proteins that were raised in TUM or NAT, respectively. **B**) Relative expression level of all immune cell-derived proteins (regardless of their glycosylation status) identified in FFPE sections of TUM vs NAT. Student's T-test (NAT, n = 5; TUM, n = 5, *$p < 0.05$).

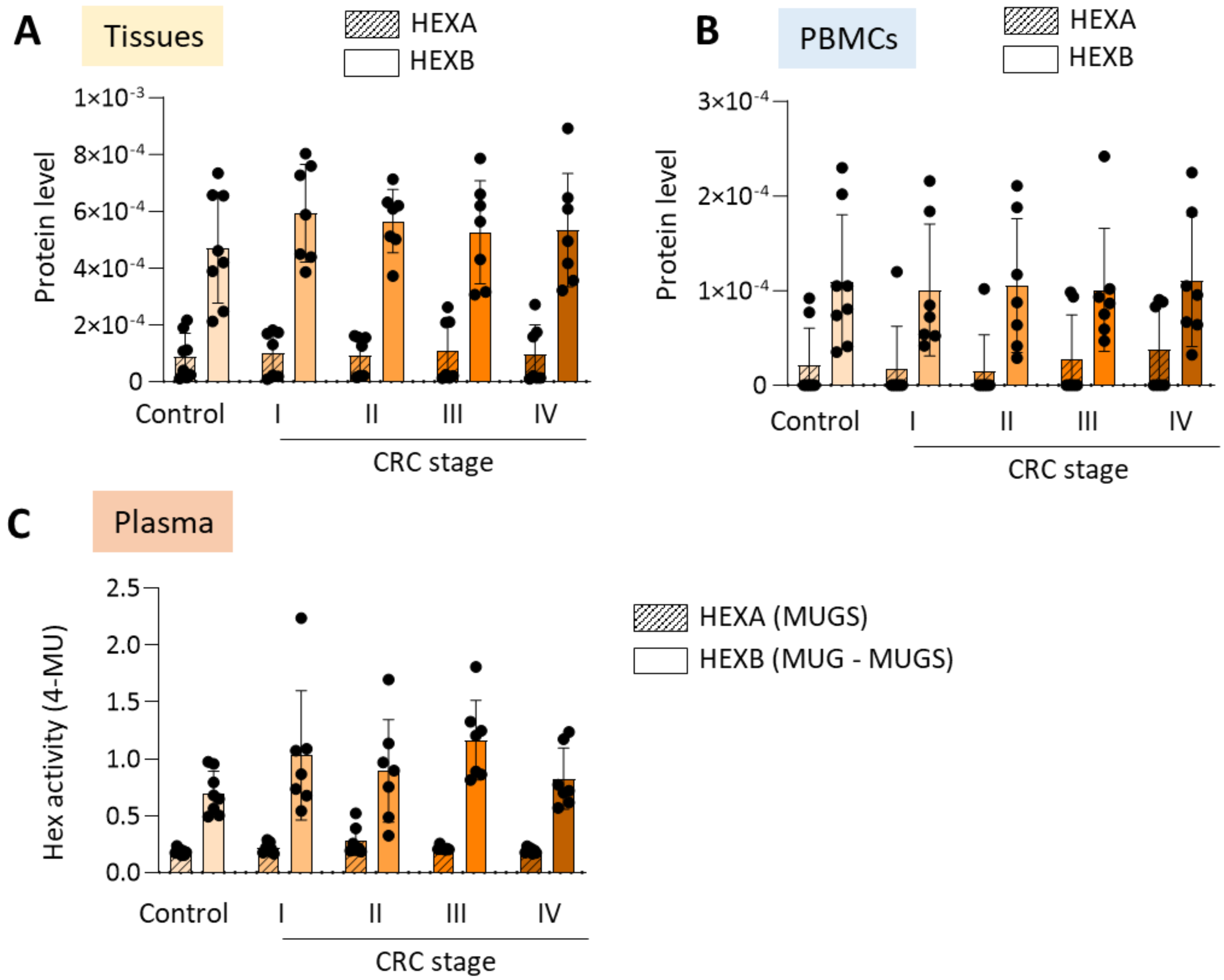

Supplementary Figure S3. **The HEXB isoenzyme form dominates in CRC**. Relative expression of the HEXA (crossed) and HEXB (open) in **A**) tumor tissues and **B**) PBMCs from CRC patients (n = 7/stage) and matching controls (n = 8) determined by quantitative proteomics. **C**) The activity of HEXA (measured by MUGS) and HEXB (measured by MUG – MUGS) isoenzyme forms in plasma from the same sample cohort.

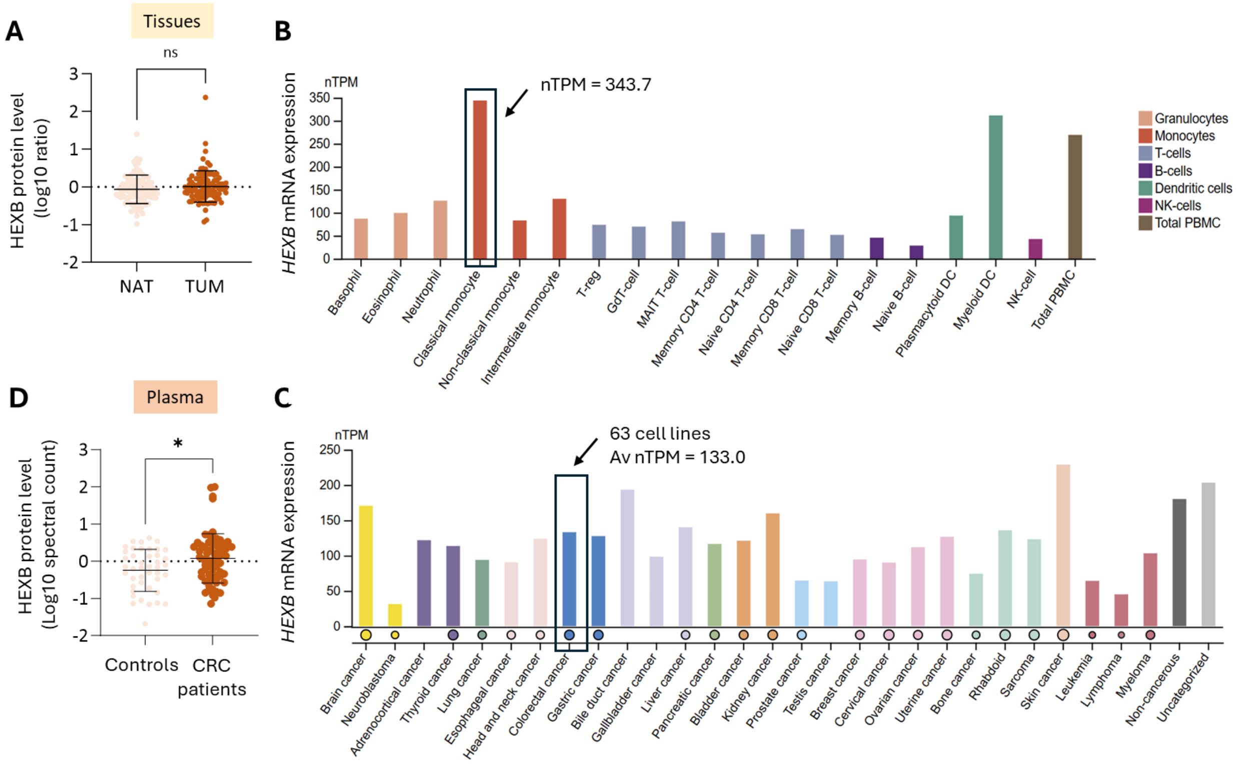

Supplementary Figure S4. HEXB protein and transcript expression in various biospecimens. **A**) Relative HEXB protein levels in paired NAT and TUM as assessed by reinterrogation of quantitative (TMT) proteomics data of 96 CRC patients obtained from CPTAC (1). Paired student's T-test, ns, non-significant $p \geq 0.05$. *HEXB* mRNA expression data (nTPM) across various **B**) immune cells including those forming the PBMC fractions and **C**) cancer cell lines (bottom) retrieved from the Human Protein Atlas. For panel B-C, the gene expression of *HEXB* in monocytes and CRC cells (average of 63 cell lines) has been indicated. **D**) HEXB protein level in plasma controls (n = 43, one outlier was identified in the control cohort using the ROUT method and was removed) and CRC plasma (n = 66, only samples from the "pre-treatment" group was used) as determined by reinterrogation of quantitative (label-free) proteomics data from Li et al. (2). Unpaired student's T-test, *$p < 0.05$.

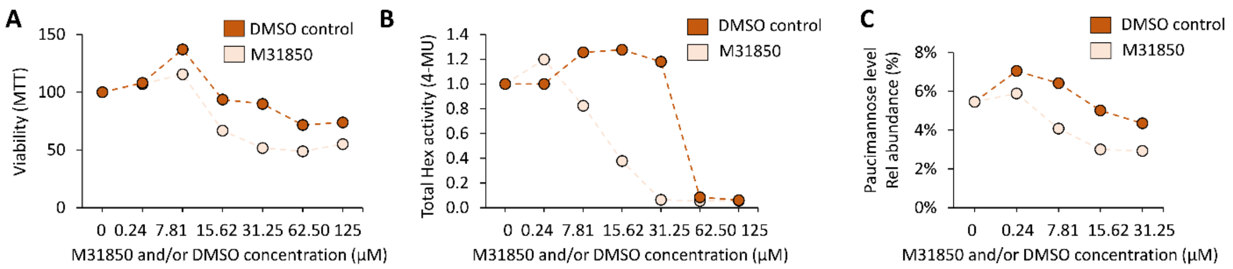

Supplementary Figure S5. Glycophenotypic impact of Hex inhibitor (M31850) and vehicle (DMSO) on CRC cells. A patient-derived CRC cell line (LIM2405) was treated for 24 h with different doses of M31850 in DMSO (0-125 µM, light orange) or with different concentrations of DMSO alone (0.24-125 µM, vehicle control, dark orange) after which several phenotypic features were monitored including: **A**) Cell viability measured using an MTT assay. **B**) Total Hex activity measured using a MUG substrate assay. **C**) Total level of paucimannosylation measured by quantitative glycomics.

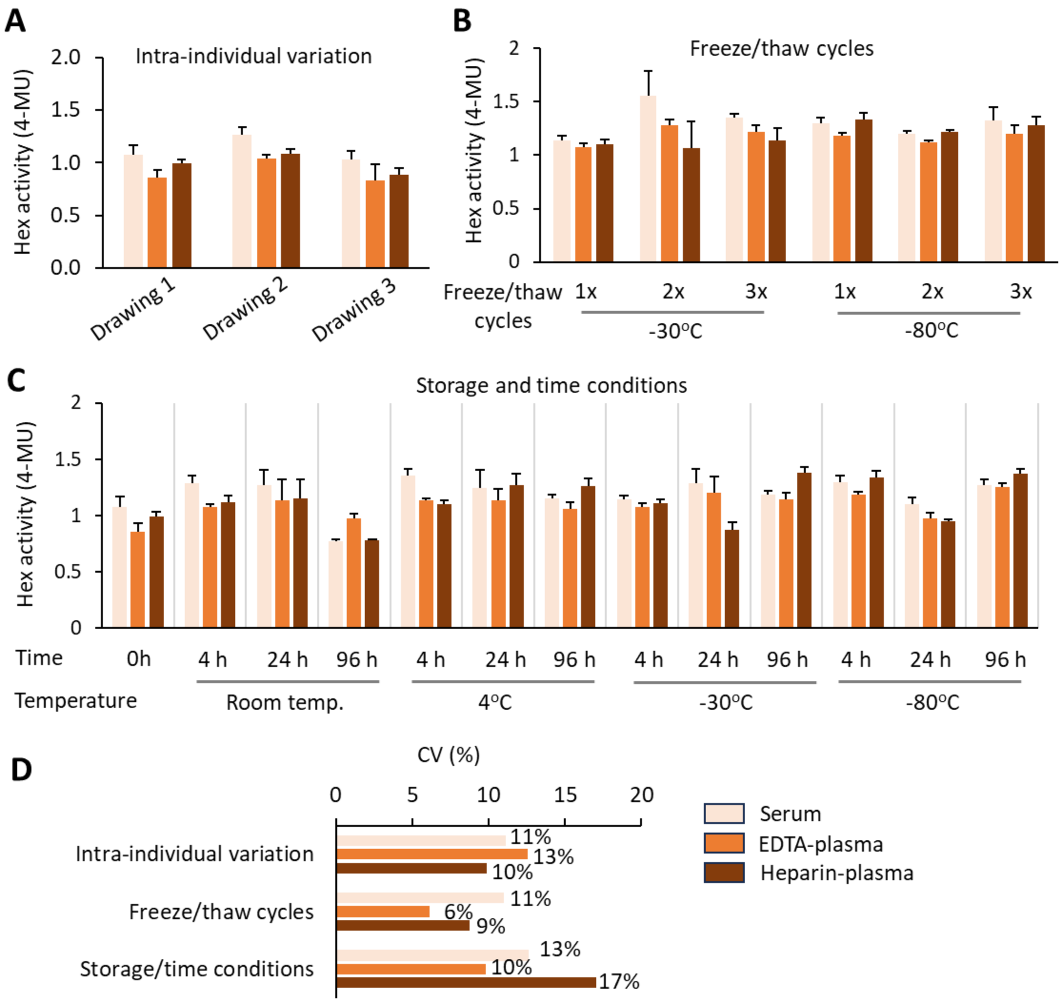

Supplementary Figure S6. Robustness of the Hex activity assay. **A**) Blood was collected from the same healthy donor over three different days (5-7 days interval). The total Hex activity (MUG) of these samples was determined in technical triplicates using identical conditions. **B**) Hex activity was measured after the sample was subjected to one, two or three freeze/thaw cycles when stored at -30°C or -80°C. **C**) The Hex activity was measured from the same biological sample (processed into serum, EDTA- and heparin-plasma) immediately after collection, or after 4 h, 24 h or 96 h of storage. Different storage conditions were compared including room temperature, 4°C, -20°C, and -80°C. **D**) Coefficient of variation (CV) across multiple assays and blood collection methods.

Supplementary Table S1. Sample cohort and experimental approaches used in this study. Gender (F: female, M: male). Experimental approaches (G: glycomics, GP: glycoproteomics, P: proteomics, H: Hex enzyme activity, IHC: immunohistochemistry, ICC: immunocytochemistry). *cellular content of epithelial cancer cells in TUM or normal epithelial cells in NAT.

**Bio-specimen**	**Condition**		**Donors** (n)	**Age** (mean ± SD)	**Gender** (F/M)	**Experimental approach** **PBMCs**	**Tissues**	**Plasma**
Matched PBMCs, tissues and plasma	CRC (stage I)		7	60 ± 19	4/3	G, GP, P, H	G, GP, P	H
	CRC (stage II)		7	69 ± 18	2/5	G, GP, P, H	G, GP, P	H
	CRC (stage III)		7	71 ± 12	3/4	G, GP, P, H	G, GP, P	H
	CRC (stage IV)		7	59 ± 9	4/3	G, GP, P, H	G, GP, P	H
Matched PBMCs and plasma	Control		8	51 ± 14	8/0	G, GP, P, H		H
Normal adjacent tissues (NAT)	Control: Paired from CRC stage I (n = 7) Unpaired from CRC stage I (n = 1)		8	60 ± 18	5/3		G, GP, P	
FFPE tissue sections (paired)	TUM	Patient 1 (stage II)	10%*	78	Male		G, IHC	
	NAT		25%*					
	TUM1	Patient 2 (stage I)	99%*	49	Male			
	NAT1		<5%*					
	TUM1	Patient 3 (stage I)	70%*	56	Female			
	NAT1		50%*					
	TUM1	Patient 4 (stage I)	60%*	-	-			
	NAT1		30%*					
FFPE tissue sections (non-paired)	TUM2	Patient 5 (stage II)	85%*	80	Male		GP, P	
	TUM2	Patient 6 (stage II)	100%*	-	-			
	TUM2	Patient 7 (stage II)	100%*	62	Male			
	TUM3	Patient 8 (stage II)	70%*	74	Male			
			70%*					
	TUM4	Patient 9 (stage II)	70%*	73	Male			
			70%*					
	NAT2	Patient 10 (stage II)	40%*	50	Male			
	NAT2	Patient 11 (stage II)	30%*	64	Male			
	NAT2	Patient 12 (stage II)	25%*	43	Male			

	NAT3	Patient 13 (stage II)	25%*<br>25%*	78	Male	
	NAT4	Patient 14 (stage II)	70%*<br>70%*	78	Male	
	TUM5	Patient 15 (stage III)	95%*	46	Female	
		Patient 16 (stage III)	90%*	70	Male	
		Patient 17 (stage III)	99%*	76	Female	
	NAT5	Patient 18 (stage III)	40%*	60	Female	
		Patient 19 (stage III)	50%*	51	Male	
		Patient 20 (stage III)	40%*	55	Female	
Plasma	CRC patients<br>Controls		302<br>78	64 ± 13<br>50 ± 18	124/178<br>51/27	H
Patient-derived CRC cell line	LIM2405		1	N/A	0/1	G, GP, P, H, ICC

Reference

1. Vasaikar, S., Huang, C., Wang, X., Petyuk, V. A., Savage, S. R., Wen, B., Dou, Y., Zhang, Y., Shi, Z., Arshad, O. A., Gritsenko, M. A., Zimmerman, L. J., McDermott, J. E., Clauss, T. R., Moore, R. J., Zhao, R., Monroe, M. E., Wang, Y. T., Chambers, M. C., Slebos, R. J. C., Lau, K. S., Mo, Q., Ding, L., Ellis, M., Thiagarajan, M., Kinsinger, C. R., Rodriguez, H., Smith, R. D., Rodland, K. D., Liebler, D. C., Liu, T., Zhang, B., and Clinical Proteomic Tumor Analysis, C. (2019) Proteogenomic Analysis of Human Colon Cancer Reveals New Therapeutic Opportunities. *Cell* 177, 1035-1049 e1019
2. Li, Y., Wang, B., Yang, W., Ma, F., Zou, J., Li, K., Tan, S., Feng, J., Wang, Y., Qin, Z., Chen, Z., and Ding, C. (2024) Longitudinal plasma proteome profiling reveals the diversity of biomarkers for diagnosis and cetuximab therapy response of colorectal cancer. *Nat Commun* 15, 980
